# Improving the health of the rural population in India through bundling WASH practices

**DOI:** 10.3389/frhs.2025.1500504

**Published:** 2025-02-19

**Authors:** Trung Thanh Nguyen, Dil Bahadur Rahut, Raja Timilsina, Manh Hung Do, Tetsushi Sonobe, Navneet Manchanda

**Affiliations:** ^1^ Institute for Environmental Economics and World Trade, Leibniz University Hannover, Hanover, Germany; ^2^ Asian Development Bank Institute (ADBI), Tokyo, Japan; ^3^ The New Delhi Office, World Bank Group, New Delhi, India

**Keywords:** sanitation, bundle, adoption, impact, disadvantaged

## Abstract

Achieving access to clean and safe water, sanitation, and hygiene (WASH) for all is one of the Sustainable Development Goals. However, most efforts to improve access to clean and safe WASH focus on a single practice, resulting in a low adoption rate and limited impact. This study analyses data from 63,732 rural households from the 76th Round of the Indian National Sample Survey in 2018 to (i) identify the factors associated with the adoption of WASH practices using logit estimations, (ii) explore adoption disparities via the Blinder-Oaxaca decomposition method, and (iii) assess the health impacts of having one vs. a combination of several, i.e., bundles of practices, using the heteroskedasticity-based instrumental variable approach. The findings reveal that (i) the wealth status of rural households and education levels of household heads are significant factors associated with the adoption, (ii) female-headed households and those belonging to scheduled castes and tribes are disadvantaged in adoption, and (iii) bundling several practices is more effective in mitigating health problems compared to single-practice adoption. Therefore, prioritizing bundled practices for impoverished households, those with lower educational attainment, female-headed households, and scheduled castes and tribes is crucial for enhancing health outcomes and alleviating the disease burden in rural India.

## 1 Introduction

Achieving universal access to clean and safe water, sanitation, and hygiene (WASH) for all is one of the United Nations Sustainable Development Goals (SDGs). The United Nations Children's Fund (1) reports that about two billion people worldwide still lack access to safe drinking water, over half of the population across the globe have inadequate sanitation arrangements, and half a billion defecate in the open. Access to clean and safe WASH practices, services, and infrastructure (hereafter referred to as WASH practices) and human health are closely interrelated, as the former can facilitate the spread of diseases and infections, adversely affecting the latter. According to the World Health Organization (WHO), inadequate and unsafe WASH practices are responsible for more than 0.8 million deaths annually, and this threatens the progress in achieving other health-related SDGs (2). Furthermore, they also exacerbates existing inequities among population groups. Physically collecting water for domestic use increases the burden on women due to the limited access to clean water at or near homes in several developing countries, and the time spent collecting water deprives women of engaging in more productive activities (3). Similarly, the threat posed by open defecation to the safety and dignity of girls and women must be considered when assessing the costs of unsafe and inadequate WASH practices. Compared to men, women are at greater risk of experiencing physical and sexual violence and developing health vulnerabilities without a well-built toilet within the household premises (4, 5). This issue is particularly concerning for pregnant women, who risk contracting infections when defecating in the open, potentially leading to prenatal complications (6). Poor sanitation also negatively affects girls' school attendance (7).

Despite numerous efforts worldwide, providing adequate and safe access to WASH practices remains a significant challenge, especially in rural areas of several developing countries (8, 9). Three critical issues require further attention: First, the adoption rate of WASH practices in rural areas of developing countries is low. Thus, it is important to identify the factors associated with adoption or non-adoption. Second, rural populations are heterogeneous, making it essential to identify population groups disadvantaged in adopting WASH practices. Third, many previous efforts focused on the impacts of a single or a limited number of WASH practices (10, 11), such as the “*No Toilet, No Bride*” campaign in India. They thus fail to capture the complementarity among adopted practices.

Against this background, this study aims (i) to identify the factors associated with the adoption of WASH practices, (ii) to determine which groups are disadvantaged in adopting WASH practices, and (iii) to analyse the impact of bundled practices on reducing sanitation-related communicable health problems in rural households. Given that India is a hotspot for inadequate and unsafe WASH practices, with rural areas particularly known for poor sanitation and hygiene (12), this study focuses on rural India. We utilize a large dataset representative of rural India from the 76th round of the National Sample Survey (NSS) conducted in 2018. Our methodology includes employing a series of logit econometric models to identify the factors associated with adoption, conducting counterfactual analyses to identify disadvantaged groups, and using a heteroscedasticity-based instrumental variable (IV) approach to account for endogeneity in impact assessment. Our findings are expected to provide valuable insights for policy responses aimed at enhancing sanitation scaling programs. These insights are relevant not only to India but also to other developing countries where unsafe WASH practices are prevalent.

The remaining parts of the paper are structured as follows. Section 2 describes the data and methodology. Section 3 presents the results. Section 4 discusses the findings. Section 5 concludes with policy implications.

## 2 Data and methodology

### 2.1 Data source

This study employs unit-level records from the National Sample Survey (NSS) 76th round undertaken in 2018 on “*Drinking Water, Sanitation, Hygiene, and Housing Condition in India*” [see (13, 14)]. The survey assesses the conditions imperative for decent and healthy living by collecting information on the availability and accessibility of clean drinking water, garbage/waste disposal, and handwashing practices. Additionally, the survey also gathers information on environmental conditions, for instance, living in an area with flies and mosquitoes, and some specific types of morbidities, which include malaria, dengue, chikungunya, and encephalitis, stomach problems like diarrhea, dysentery, cholera and jaundice, skin, and other diseases.^1^ The survey covers the whole country except the villages in Andaman and Nicobar Islands which are difficult to access. A stratified two-stage sampling procedure was adopted, leading to a total sample of 106,838 households (63,736 rural and 43,102 urban). Sampling weights were calculated for adjustments of differential selection probabilities. As inadequate and unsafe WASH practices and their associated health problems are more prevalent in rural India, this study uses the data from rural households. Among 63,736 rural households that were surveyed, we excluded four households due to missing information across the sections that we use for our analysis. Therefore, the final sample in this study includes 63,732 rural households in India. The WASH practices recorded in the data include (i) drinking water from a tap, (ii) non-drinking water from a tap, (iii) treated drinking water, (iv) handwashing with soap before a meal, (v) having toilets (exclusive use of the household), (vi) having bathrooms (exclusive use of the household), (vii) having underground or covered pucca drainage, and (viii) arranged garbage collection. The health problems related to inadequate and unsafe WASH practices include (i) skin diseases of household members (skin problems), (ii) living in areas with flies and mosquitoes (fly and mosquito problems), (iii) stomach problems of household members (stomach problems), (iv) malaria, dengue, chikungunya, and encephalitis problems of household members (malaria problems), and (v) other WASH-related problems of household members.

### 2.2 Methodology

#### 2.2.1 Identifying the factors associated with WASH adoption using logit regression

We divide our sampled households into the following groups: (i) non-adopters (adopting none of the eight practices), (ii) adopters of only one practice, and (iii) adopters of more than one practice, i.e., bundling two, bundling three, bundling four, bundling five, bundling six, and bundling more than six. Our first step is to identify the factors associated with the adoption of only one specific sanitation practice as follows:

(1) $$SA\_single_{i}=\;\varphi +\beta X_{i}+\gamma S_{k}+\varepsilon_{i}$$

where *SA_single* = 1 if household *i* in state *k* adopted only a specific practice, and 0 if it is a non-adopter.

Next, we identify the factors associated with the adoption of different bundles of practices (from adopting one practice to adopting more than six) as follows:

(2) $$SA\_bundle_{i}=\;\vartheta +\rho X_{i}+\sigma S_{k}+{\text{£}}_{i}$$

where, for adopting one practice *SA_bundle* = 1 if household *i* in state *k* adopted one practice, and 0 if the household is a non-adopter; for adopting two practices *SA_bundle* = 1 if household *i* in state *k* adopted two practices, and 0 if it is a non-adopter. This is similar to bundling three, four, five, six or more than six practices.

In Equations 1, 2 $X_{i}$ are the vector of household characteristics which include (i) demographic characteristics of the household head and the household (i.e., age, gender, and education of household head; household size, number of males, number of children, and number of elders), (ii) wealth status (i.e., monthly per capital expenditure), (iii) social groups (i.e., scheduled castes or scheduled tribes), and (iv) religions (i.e., Hindu, Islam, Christianity, Sikhism, and Buddhism); $S_{k}$ is the dummies for states; and $\varepsilon_{i}$, and ${\text{£}}_{i}$ are the error terms of Equations 1, 2, respectively (see more information on the variables in Supplementary Table S1). Since there are several independent varibles, there is a potential of multi-collinearity. We checked this issue, and the results of the variance inflation factor (VIF) values (in Supplementary Table S2) show no sign of this problem. We run logistic regressions for Equations 1, 2 with sample weights and cluster our standard errors at the district level to obtain robust standard errors and prevent auto-correlation (15).

#### 2.2.2 Determining disadvantaged groups with counterfactual analysis for gender and scheduled castes and tribes

We employ the Blinder-Oaxaca decomposition method (16, 17) to examine the differences in the adoption of WASH practices with regard to (i) the gender of the household head (male vs. female), and (ii) if the household belongs to the scheduled castes or tribes (scheduled castes or tribes vs. the rest). This statistical technique is selected as it allows us to quantify and decompose the effects of the factors contributing to disparities in outcome variables between two groups, for example differences in health outcomes between male- and female-headed households. Given two groups, *A* and *B*, the difference in the adoption between the two groups for outcome variable *Y* can be specified as in Equation 3:

(3) $$D=E(Y_{A})-E(Y_{B})$$

where *E*(*Y*) is the expected value of outcome *Y*, which can be estimated as follows:

(4) $$Y_{i}=X_{i}\beta_{i}+\epsilon_{i},\;E(\epsilon_{i})=0;i\;\in (A,\;B)$$

In Equation 4, $X_{i}$ is a vector of household *i*'s characteristics and $\epsilon_{i}$ is the error term. The difference of mean outcome can be defined as the difference in the linear estimation at the group-specific means of the regressors as in Equation 5:

(5) $$D=E(Y_{A})-E(Y_{B})=E(X_{A}{)}^{\prime }\beta_{A}-E(X_{B}{)}^{\prime }\beta_{B}$$

Therefore, the difference between the two groups in the overall outcome difference is:

(6) $$D=\{E(X_{A})-E(X_{B}){\}}^{\prime }\beta_{B}+E(X_{B}{)}^{\prime }(\beta_{A}-\beta_{B})+\{E(X_{A})-E(X_{B}){\}}^{\prime }(\beta_{A}-\beta_{B})$$

From Equation 6, the “threefold” decompositions are as in Equation 7:

(7) D=E+C+I

where:

(7a) $$E=\{E(X_{A})-E(X_{B}){\}}^{\prime }\beta_{B}$$

(7b) $$C=E(X_{B}{)}^{\prime }(\beta_{A}-\beta_{B})$$

(7c) $$I=\{E(X_{A})-E(X_{B)}{\}}^{\prime }(\beta_{A}-\beta_{B})$$

Equations 7a–c demonstrate the endowments effect (*E*), the contribution of differences in the coefficients (*C*), and the differences in endowments and coefficients that exist simultaneously between the two groups (*I*), respectively. In particular, the endowments effect reflects the change of the probabilities of adopting WASH practices in one group if they had the same characteristics as the other group (i.e., between male- and female-headed households or between scheduled castes/tribes and the rest). The contribution of coefficients captures the change of the probabilities of adopting WASH practices in one group when applying the coefficients of the other group. The differences in endowments and coefficients (as the interaction) accounts for the fact that differences in endowments and coefficients exist simultaneously between the two groups. We employ Jann (18)'s Stata codes to implement this procedure. The sample weights are also used as the analytical weights in the estimations of the Blinder-Oaxaca decomposition model.

#### 2.2.3 Evaluating the impacts of sanitation practices on reducing health problems

We evaluate the impacts of adopting different bundles of WASH practices on the appearance of WASH-related health problems. More specifically,

(8) $$H_{i}=\;\phi +\chi SA_{i}+\psi X_{i}+\varepsilon_{i}$$

In Equation 8, $H_{i}$ is a dummy variable indicating if (a member of) household *i* suffered from a specific category of health problems (1 = yes and 0 = otherwise); $SA_{i}$ captures the adopted practices of household *i* and can be either *SA_single* (as in Equation 1) or *SA_bundle* (as in Equation 2).

As $SA_{i}$ is determined by $X_{i}$ as in Equations 1, 2, it is endogenous in Equation 8. This violates the exogeneity assumption of the Gauss-Markov theorem and leads to biased estimates if the endogeneity is unaddressed. Therefore, we employ the heteroskedasticity-based instrumental variable (IV) approach suggested by Lewbel (19) to address the endogeneity in our impact assessments. Assuming a general form of the adoption function as

(9) $$SA_{i}=\;\Psi +\Omega^{\prime }X_{i}+\zeta_{i}$$

which indicates the endogeneity of $SA_{i}$. The heteroscedasticity-based IV approach assumes that there is an existence of heteroscedasticity in $\zeta_{i}$ (and $\;SA_{i}$). The usual assumptions are specified as in Equation 10:

(10) $$Cov(X_{i},\varepsilon_{i})=Cov(X_{i},\zeta_{i})=Cov(X_{i},\varepsilon_{i}\zeta_{i})=0$$

The heteroskedasticity-based method additionally assumes the heteroscedasticity in Equation 9 as in Equation 11:

(11) $$Cov(X_{i},\zeta_{i}^{2})\neq 0$$

Lewbel (19) suggests using the $[X_{i}-E(X_{i})]{\hat{\zeta }}_{i}$ as an internal IV for $SA_{i}$ in regressing Equation 8, where ${\hat{\zeta }}_{i}$ is the predicted residuals obtained from estimating Equation 9 without the inclusion of $SA_{i}$. The IV is considered as a valid instrument since $[X_{i}-E(X_{i})]{\hat{\zeta }}_{i}$ is uncorrelated with $\varepsilon_{i}$ in Equation 8. This method can be applied to both binary and continuous outcomes and regressors. Besides, the sample weights are again used as the analytic weights in the estimations of impacts. We also carry out post-estimation tests to validate the IVs in our estimation, namely the under-identification test [a LM test based on Kleibergen and Paap (20)], the weak identification test using Kleibergen-Paap rk Wald F statistics, and the over-identification test based on the Hansen J statistic test.^2^ In addition, we check the robustness of our results by employing the propensity score matching (PSM) with the nearest neighbor matching. After estimating the propensity scores, we carry out covariate balancing tests to see whether the estimations of the scores can balance the covariate characteristics of the observations between the treated and control groups.^3^ All estimations are undertaken using the statistical program Stata 15/IC.

## 3 Results

### 3.1 Descriptive statistics

**Figure 1:**
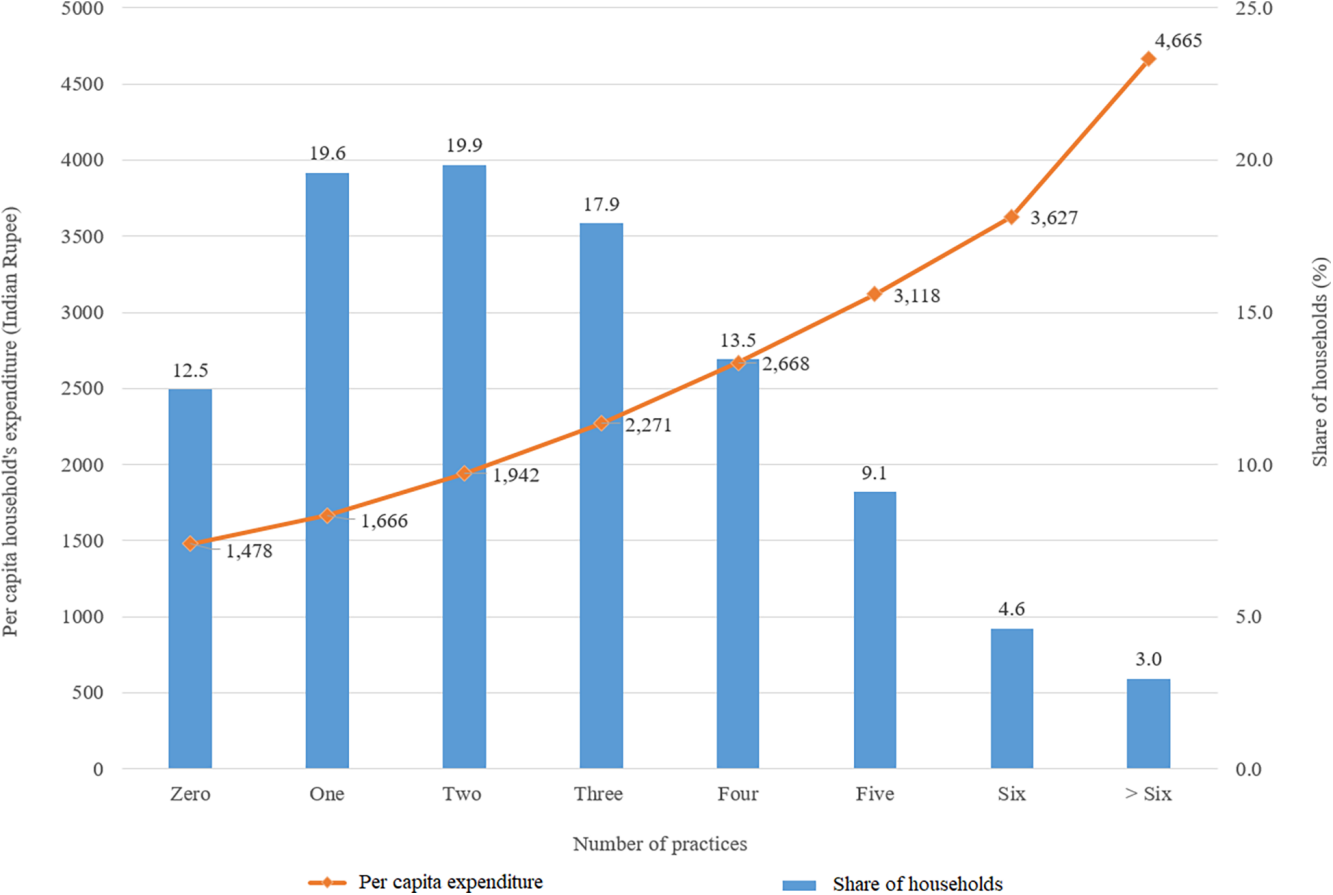
Distribution and monthly per capita consumption expenditure and share of rural households adopting different numbers of WASH practices.

**Figure 2:**
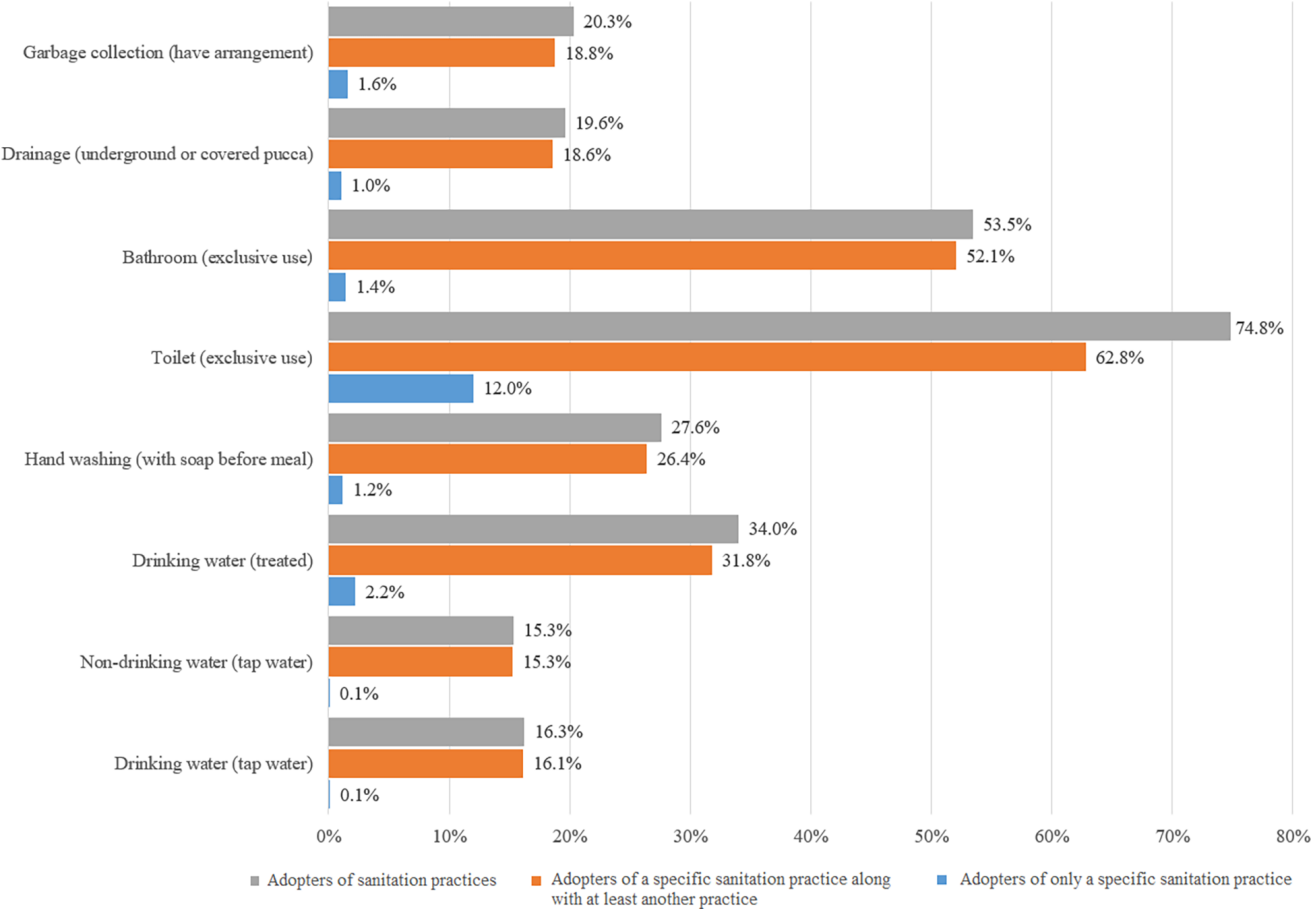
Distribution of WASH practices adopted by rural households.

Figure 1 shows that 12.5% of the households do not have any WASH practices, and 3% of the sampled households have more than six practices. The majority of the households have 1–3 practices. The per capita expenditure trend line is positive and increases with the number of practices. Figure 2 shows that 74.8% of households in rural India have exclusive-use toilets, and 53.5% have exclusive-use of bathrooms. However, the share of rural households with garbage collection, drainage, drinking, and non-drinking water from taps services is less than 20%.

The upper panel of Table 1 presents the descriptive summary of WASH-related communicable health problems that the households faced in the last 365 days. Fly and mosquito problems appear to be the most popular. Overall, non-adopting households have a higher share of health problems than adopting households. About 12.7% of the households are female-headed, and the share of households with female heads without any WASH practices is 13.8%. Households from the scheduled castes or tribes adopt fewer WASH practices. The lower panel of Table 1 summarises the characteristics of sampled households.

**Table 1:** Descriptive statistics of household's health problems and characteristics.

Variables	Whole sample (*n* = 63,732)	Without WASH practices (*n* = 7,955)	With only one WASH practice (*n* = 12,483)^c^	With more than one WASH practice (*n* = 43,294)^d^
Health problems				
Share of households having skin problems (%)	7.51	10.02	9.24^b^	6.34***^,^^b^
Share of households having fly and mosquito problems (%)	48.62	59.58	55.37***^,^^b^	43.81***^,^^b^
Share of households having stomach problems (%)	15.64	20.38	17.60***^,^^b^	13.89***^,^^b^
Share of households having malaria problems (%)	11.19	14.39	12.29***^,^^b^	10.08***^,^^b^
Share of households having other problems (%)	32.97	39.73	36.59**^,^^b^	30.19***^,^^b^
Household's characteristics				
Monthly expenditure per capita (INR)	1,900.23	1,414.02	1,582.93***^,^^a^	2,119.97***^,^^a^
	(1,014.72)	(521.58)	(591.24)	(1,139.39)
Share of female heads (%)	12.73	13.81	12.25***^,^^b^	12.64**^,^^b^
Age head (years)	47.25	45.22	45.69***^,^^a^	48.24***^,^^a^
	(13.44)	(13.32)	(13.28)	(13.42)
Household size (persons)	4.53	4.55	4.48^a^	4.54***^,^^a^
	(2.12)	(2.09)	(2.07)	(2.14)
No. of males (persons)	2.32	2.317	2.304^a^	2.33***^,^^a^
	(1.31)	(1.308)	(1.292)	(1.31)
No. of children (children)	1.41	1.296	1.36***^,^^a^	1.46***^,^^a^
	(0.94)	(0.888)	(0.91)	(0.95)
No. of elders (persons)	0.40	0.34	0.35^a^	0.44***^,^^a^
	(0.67)	(0.63)	(0.63)	(0.70)
Share of illiterate heads (%)	34.69	50.56	40.89***^,^^b^	28.93***^,^^b^
Share of heads with high school education (%)	6.35	3.03	4.09***^,^^b^	7.89***^,^^b^
Share of heads with diploma certificate (%)	0.73	0.29	0.23^b^	1.00***^,^^b^
Share of heads with university education and above (%)	4.74	1.24	2.03***^,^^b^	6.46***^,^^b^
Share of households belonging to scheduled tribes (%)	12.23	16.61	15.44^b^	10.13^b^
Share of households belonging to scheduled castes (%)	21.95	29.99	25.83***^,^^b^	18.79***^,^^b^
Share of Hindu households (%)	82.31	87.29	82.19***^,^^b^	81.21***^,^^b^
Share of Islam households (%)	11.9	9.59	14.90***^,^^b^	11.41**^,^^b^
Share of Christianity households (%)	2.53	1.24	1.31^b^	3.24***^,^^b^
Share of Sikhism households (%)	1.58	0.22	0.21^b^	2.35***^,^^b^
Share of Buddhism households (%)	0.48	0.08	0.22**^,^^b^	0.66***^,^^b^

Standard deviation in parentheses.

^a^ Two-sample *t*-test.

^b^ Pearson chi-squared statistic corrected for the survey design with the second-order correction of Rao and Scott (21).

^c^ Between no practices and one practice.

^d^ Between no practices and more than one practice.

^∗∗∗^ *p* < 0.01, ^∗∗^*p* < 0.05, ^∗^*p* < 0.1.

### 3.2 Factors associating with the adoption of WASH practices

Table 2 documents the estimated results of Equation 1 (logit estimations) and shows the marginal effects of the factors associated with the adoption of one specific practice. The marginal effects allow us to interpret a coefficient as percentage change. The coefficient of household's monthly per capita expenditure is positive and significant in most estimations except for drinking water from the tap and treated drinking water. In particular, if the monthly expenditure per capita increases by 1%, the households are more likely to adopt wash hand before meal with soap by 0.07%, toilet (exclusive use) by 0.19%, bathroom (exclusive use) by 0.09%, drainage (under-ground or covered pucca) by 0.04%, and arranged garbage collection by 0.05%. The coefficient of female-headed household is positive but insignificant except for drainage (underground or covered pucca). The age of the household head is insignificant except for the toilet (exclusively for household use), which is positive, indicating that households with an older head are more likely to adopt toilets. Households with large family members are more likely to adopt toilets and bathrooms exclusively for household use and drainage (underground or covered pucca), which may be due to the availability of labor force to maintain these practices. Households with illiterate heads are less likely to adopt the practices, such as handwashing with soap before meals, toilets, and bathrooms, compared to household heads with at least a primary level of education. The high school and university levels of education are positive and significant only for toilet use. Households belonging to the scheduled tribes are less likely to adopt or have a toilet and drainage (underground or pucca-covered) sanitation infrastructure, while only drainage is significant for scheduled caste.

**Table 2:** Factors associating with adoption of only one WASH practice (logit—marginal effect).

	Drinking water (tap)	Treated drink- water	Wash hand before meal with soap	Toilet (exclusive use)	Bathroom (exclusive use)	Drainage (under-ground or covered pucca)	Arranged garbage collection
	(1)	(2)	(3)	(4)	(5)	(6)	(7)
Monthly expenditure per capita (ln)	0.005	0.012	0.066***	0.191***	0.086***	0.043**	0.045***
	(0.005)	(0.020)	(0.018)	(0.026)	(0.014)	(0.017)	(0.014)
Female head	−0.008	−0.011	0.013	0.004	0.004	0.019**	0.004
	(0.006)	(0.009)	(0.010)	(0.015)	(0.013)	(0.010)	(0.011)
Age head	0.000	−0.000	−0.000	0.002***	0.000	0.000	−0.000
	(0.000)	(0.000)	(0.000)	(0.001)	(0.000)	(0.000)	(0.000)
Household size	0.001	0.004	−0.001	0.010**	0.008***	0.007***	0.003
	(0.001)	(0.003)	(0.003)	(0.004)	(0.002)	(0.002)	(0.003)
No. of males	−0.002	−0.005	0.008*	−0.001	0.004	−0.005	0.003
	(0.002)	(0.006)	(0.004)	(0.007)	(0.005)	(0.004)	(0.005)
No. of children	−0.001	−0.000	0.002	0.016**	0.007	0.005	0.001
	(0.003)	(0.006)	(0.005)	(0.006)	(0.007)	(0.005)	(0.005)
No. of elders	−0.002	0.000	0.005	0.005	−0.002	0.001	0.001
	(0.004)	(0.008)	(0.007)	(0.008)	(0.008)	(0.007)	(0.009)
Illiterate head	−0.004	−0.007	−0.021**	−0.088***	−0.020**	−0.028***	−0.009
	(0.003)	(0.008)	(0.009)	(0.013)	(0.010)	(0.007)	(0.009)
High school head	0.006	−0.024	0.024	0.064***	−0.009	0.008	−0.030
	(0.009)	(0.028)	(0.018)	(0.020)	(0.015)	(0.012)	(0.023)
Diploma head	0.006	−0.092	−0.100	−0.089	−0.030	−0.068	−0.017
	(0.011)	(0.118)	(0.073)	(0.084)	(0.058)	(0.054)	(0.058)
Uni and above head	0.008	0.045	0.026	0.136***	0.008	−0.011	0.001
	(0.008)	(0.033)	(0.020)	(0.034)	(0.029)	(0.024)	(0.026)
Scheduled tribes	−0.010	0.015	−0.027	−0.051**	−0.026	−0.081***	−0.037
	(0.007)	(0.015)	(0.017)	(0.021)	(0.019)	(0.025)	(0.023)
Scheduled castes	0.006	0.030**	−0.011	−0.012	−0.017	−0.023**	0.009
	(0.004)	(0.014)	(0.011)	(0.016)	(0.012)	(0.010)	(0.012)
Hindu	−0.010	−0.065*	−0.013	0.069*	0.009	0.016	0.053
	(0.011)	(0.034)	(0.028)	(0.040)	(0.027)	(0.025)	(0.037)
Islam	−0.001	−0.062	−0.009	0.166***	−0.000	−0.003	0.062*
	(0.011)	(0.045)	(0.030)	(0.052)	(0.031)	(0.027)	(0.035)
Christianity	−0.015	−0.047	0.015	0.120	0.014	−0.040	0.006
	(0.018)	(0.041)	(0.070)	(0.078)	(0.050)	(0.074)	(0.036)
Sikhism	0.000	−0.209***	−0.120**	0.157	0.059	0.000	0.000
	(0.000)	(0.078)	(0.060)	(0.145)	(0.084)	(0.000)	(0.000)
Buddhism	0.000	−0.041	−0.219**	0.174	0.067	0.051	0.000
	(0.000)	(0.070)	(0.099)	(0.134)	(0.054)	(0.062)	(0.000)
State dummies	Yes	Yes	Yes	Yes	Yes	Yes	Yes
Number of observations	8,030	9,362	8,710	15,602	8,858	8,615	8,944
Pseudo *R*^2^	0.311	0.334	0.062	0.145	0.164	0.156	0.214

Robust standard errors clustered at district levels in parentheses; estimation on non-drinking water (tap) is excluded due to insufficient observations; the number of observations includes the non-adopters plus the adopters of each specific practice; ∗∗∗*p* < 0.01, ∗∗*p* < 0.05, ∗*p* < 0.1.

Table 3 documents the estimated results of Equation 2 (logit estimations) and shows the marginal effects of the factors associating with the adoption of different bundles of WASH practices. The monthly per capita expenditure, age of the household head, household size, and the number of children under 15 years are positive and significant. In particular, the coefficients of the monthly per capita expenditure indicate that if the expenditure increases by 1%, the households are more likely to adopt bundling two practices by 0.28%, bundling three practices by 0.29%, bundling four practices by 0.25%, bundling five practices by 0.19%, bundling six practices by 0.14%, and bundling more than six practices by 0.11%. Similar to the results reported in Table 2, households with illiterate heads are less likely to adopt a WASH bundle compared to households with primary school-completed heads. Households in the scheduled castes and tribes are less likely to adopt a WASH bundle.

**Table 3:** Factors associating with adoption of different bundles of WASH practices (logit—marginal effects).

	Bundling two	Bunding three	Bundling four	Bundling five	Bundling six	Bundling more than six
	(2)	(3)	(4)	(5)	(6)	(7)
Monthly expenditure per capita (ln)	0.279***	0.290***	0.247***	0.193***	0.139***	0.107***
	(0.022)	(0.021)	(0.020)	(0.014)	(0.010)	(0.007)
Female head	0.018	0.060***	0.035***	0.014	0.004	0.016**
	(0.015)	(0.014)	(0.012)	(0.009)	(0.009)	(0.008)
Age head	0.002***	0.002**	0.002***	0.001**	0.001***	0.001***
	(0.001)	(0.001)	(0.001)	(0.001)	(0.000)	(0.000)
Household size	0.021***	0.021***	0.019***	0.015***	0.011***	0.008***
	(0.003)	(0.003)	(0.003)	(0.003)	(0.002)	(0.002)
No. of males	−0.004	−0.005	−0.010***	−0.007	−0.011**	−0.006*
	(0.005)	(0.005)	(0.004)	(0.005)	(0.005)	(0.004)
No. of children	0.029***	0.037***	0.029***	0.017***	0.021***	0.009**
	(0.006)	(0.006)	(0.004)	(0.004)	(0.004)	(0.004)
No. of elders	0.010	0.020**	0.014*	0.006	0.008*	−0.001
	(0.008)	(0.009)	(0.008)	(0.007)	(0.004)	(0.004)
Illiterate head	−0.127***	−0.130***	−0.108***	−0.093***	−0.066***	−0.062***
	(0.013)	(0.010)	(0.008)	(0.009)	(0.008)	(0.008)
High school head	0.103***	0.114***	0.085***	0.061***	0.044***	0.037***
	(0.018)	(0.014)	(0.014)	(0.017)	(0.011)	(0.011)
Diploma head	0.031	0.118**	0.065**	0.040	0.062***	0.035*
	(0.061)	(0.055)	(0.033)	(0.026)	(0.021)	(0.018)
Uni and above head	0.185***	0.233***	0.186***	0.145***	0.127***	0.073***
	(0.030)	(0.024)	(0.018)	(0.018)	(0.015)	(0.010)
Scheduled castes	−0.114***	−0.103***	−0.096***	−0.117***	−0.098***	−0.065***
	(0.021)	(0.021)	(0.017)	(0.020)	(0.016)	(0.018)
Scheduled tribes	−0.054***	−0.062***	−0.044***	−0.027**	−0.023***	−0.012
	(0.015)	(0.012)	(0.013)	(0.011)	(0.007)	(0.008)
Hindu	0.026	0.011	0.019	0.001	0.042*	0.010
	(0.044)	(0.042)	(0.029)	(0.030)	(0.025)	(0.018)
Islam	0.092*	0.076	0.075**	0.015	0.065***	0.022
	(0.051)	(0.047)	(0.031)	(0.036)	(0.024)	(0.018)
Christianity	0.114	0.023	0.133*	0.013	0.037	0.010
	(0.072)	(0.066)	(0.079)	(0.039)	(0.025)	(0.017)
Sikhism	0.006	0.138*	0.167**	0.049	0.035	−0.015
	(0.082)	(0.078)	(0.071)	(0.043)	(0.037)	(0.021)
Buddhism	0.197	0.201*	0.127*	−0.012	0.063	0.012
	(0.125)	(0.110)	(0.067)	(0.078)	(0.060)	(0.041)
State dummies	Yes	Yes	Yes	Yes	Yes	Yes
Number of observations	20,608	19,387	16,528	13,753	10,902	9,846
Pseudo *R*^2^	0.198	0.352	0.510	0.617	0.690	0.729

Robust standard errors clustered at district levels in parentheses; The number of observations includes the non-adopters plus the adopters of each group; ∗∗∗*p* < 0.01, ∗∗*p* < 0.05, ∗*p* < 0.1.

### 3.3 Disadvantaged groups in adoption of WASH practices

Table 4 presents the results of the counterfactual analyses between male and female-headed households using the Blinder Oaxaca non-linear decomposition method. This method allows us to calculate the gender gap in the adoption of WASH practices. The results show that the gaps are significant. In particular, the probabilities of adopting one to more than six practices appear to be higher in male-headed households. The gaps between male- and female-headed households are about 3.4% in adopting one practice and 5.2% in adopting two practices. Further, the probability of female-headed households adopting more than five practices would increase significantly if they had the same endowment as male-headed households. We also perform the counterfactual analysis between female-headed and male-headed households for (i) adopting only one specific practice, and (ii) adopting multiple bundles.^4^ Results of these additional analyses show that female-headed households are consistently disadvantaged and largely due to differences in endowment. Therefore, female-headed households should be targeted in national sanitation programs for rural development.

**Table 4:** Counterfactual analysis of adopting different bundles of WASH practices between female- and male-headed households.

	Probabilities of adopting						
	One practice	Bundling two	Bunding three	Bundling four	Bundling five	Bundling six	Bundling more than six
	(1)	(2)	(3)	(4)	(5)	(6)	(7)
Differential							
Male-headed	0.600***	0.583***	0.536***	0.445***	0.371***	0.226***	0.138***
	(0.011)	(0.014)	(0.024)	(0.039)	(0.048)	(0.038)	(0.030)
Female-headed	0.567***	0.531***	0.520***	0.449***	0.354***	0.201***	0.129***
	(0.015)	(0.017)	(0.032)	(0.053)	(0.059)	(0.040)	(0.030)
Difference	0.034***	0.052***	0.016	−0.004	0.018	0.025	0.009
	(0.005)	(0.008)	(0.016)	(0.025)	(0.026)	(0.016)	(0.014)
Decomposition							
Endowments^a^	0.037***	0.071***	0.084***	0.070***	0.060**	0.057***	0.069***
	(0.005)	(0.008)	(0.016)	(0.025)	(0.026)	(0.017)	(0.018)
Coefficients^b^	−0.004***	−0.017***	−0.070***	−0.068***	−0.034***	−0.027***	−0.037***
	(0.000)	(0.001)	(0.002)	(0.003)	(0.005)	(0.007)	(0.007)
Interaction^c^	0.000	−0.002*	0.003	−0.005	−0.009***	−0.005	−0.023***
	(0.001)	(0.001)	(0.004)	(0.004)	(0.003)	(0.005)	(0.006)
Number of observations	20,438	20,608	19,387	16,528	13,753	10,902	9,846

Robust standard errors clustered at state levels in parentheses.

^a^ The change of the probabilities of adoption in female-headed households if they had the same characteristics as male-headed households. For example, the value of 0.037 in column (1) means: the probability of adopting one practice would increase by 3.7% if female-headed households had the same characteristics as male-headed households.

^b^ The change of the probabilities of adoption in female-headed households when applying the coefficients of male-headed households to female-headed households. For example, the value of −0.004 in column (1) means: the probability of adopting one practice would decrease by 0.4% when applying male-headed households' coefficients to female-headed households.

^c^ The interaction term accounting for the fact that differences in endowments and coefficients exist simultaneously between (a) and (b).

∗∗∗ *p* < 0.01, ∗∗*p* < 0.05, ∗*p* < 0.1.

Results of the counterfactual analyses between households in the scheduled castes or tribes and the rest of the households presented in Table 5. The results show a significantly lower adoption of WASH practices in households in the scheduled castes or tribes as compared to the rest of the sample. More specifically, it is 5.2% in adopting one practice, 13.2% in adopting two practices, 16.9% in adopting three practices, 20.4% in adopting four practices, 23.3% in adopting five practices, 18.3% in adopting six practices, and 12.7% in adopting more than six practices. Additional analyses using the Blinder Oaxaca decomposition method also show that the households in scheduled castes and tribes are consistently disadvantaged.^5^

**Table 5:** Counterfactual analysis of adopting different bundles of WASH practices between households in the scheduled castes (SC) or scheduled tribes (ST) and other households.

	Probabilities of adopting						
	One practice	Bundling two	Bunding three	Bundling four	Bundling five	Bundling six	Bundling more than six
	(1)	(2)	(3)	(4)	(5)	(6)	(7)
Differential							
Other households	0.619***	0.629***	0.598***	0.523***	0.458***	0.297***	0.191***
	(0.013)	(0.012)	(0.021)	(0.040)	(0.053)	(0.048)	(0.040)
ST or SC households	0.566***	0.497***	0.429***	0.319***	0.224***	0.115***	0.064***
	(0.012)	(0.020)	(0.033)	(0.039)	(0.035)	(0.024)	(0.016)
Difference	0.052***	0.132***	0.169***	0.204***	0.233***	0.183***	0.127***
	(0.006)	(0.010)	(0.016)	(0.018)	(0.030)	(0.030)	(0.029)
Decomposition							
Endowments^a^	0.040***	0.059***	0.075***	0.141***	0.147***	0.091***	0.047***
	(0.005)	(0.010)	(0.015)	(0.018)	(0.026)	(0.019)	(0.015)
Coefficients^b^	−0.001	0.063***	0.073***	0.061***	0.065***	0.045***	0.020
	(0.004)	(0.004)	(0.006)	(0.005)	(0.008)	(0.013)	(0.014)
Interaction^c^	0.014***	0.009***	0.021***	0.002	0.021*	0.046***	0.059***
	(0.004)	(0.003)	(0.006)	(0.006)	(0.011)	(0.010)	(0.011)
Number of observations	20,438	20,608	19,387	16,528	13,753	10,902	9,846

Robust standard errors clustered at state levels in parentheses.

^a^ The change of the probabilities of adoption in the households in ST or SC groups if they had the same characteristics as other households. For example, the value of 0.040 in column (1) means: the probability of adopting one practice in ST or SC households would increase by 4.0% if ST or SC households had the same characteristics as other households.

^b^ The change of the probabilities of adoption in the households in ST or SC social groups when applying the coefficients of other households to the households in ST or SC group.

^c^ The interaction term accounting for the fact that differences in endowments and coefficients exist simultaneously between (a) and (b). For example, the value of 0.014 in column (1) means the difference between the other households and ST or SC households in the endowments and coefficient contributes about 1.4% to the difference in the adoption of one practice.

∗∗∗ *p* < 0.01, ∗*p* < 0.1.

### 3.4 Impact of WASH practices on health problems

Table 6 summarizes the impacts of adopting only one specific WASH practice on WASH-related health problems and demonstrates that the impacts are mainly insignificant. Only a small number of individual WASH practices (non-drinking water, bathroom, and drainage-underground or covered pucca) significantly affect a small number of WASH-related health problems. This means adopting only a particular WASH practice does not significantly reduce the appearance of WASH-related health problems.

**Table 6:** Impacts of adopting only one specific sanitation practice on health problems.

	Skin problems	Fly and mosquito problems	Stomach problems	Malaria problems	Other problems
	(1)	(2)	(3)	(4)	(5)
Drinking water (tap)	−0.050	0.031	−0.067	−0.061	−0.187*
	(0.035)	(0.111)	(0.090)	(0.053)	(0.095)
Non-drinking water (tap)	−0.099***	0.163	−0.004	0.043	−0.129
	(0.027)	(0.101)	(0.053)	(0.080)	(0.083)
Treated drinking water	−0.064	−0.093	0.156	−0.237	−0.037
	(0.087)	(0.193)	(0.140)	(0.189)	(0.099)
Wash hand before meal with soap	0.097	0.003	−0.085	−0.017	−0.250
	(0.084)	(0.173)	(0.127)	(0.043)	(0.237)
Toilet (exclusive use)	0.164	0.154	0.281	0.184	0.243
	(0.223)	(0.277)	(0.279)	(0.308)	(0.285)
Bathroom (exclusive use)	−0.080	−0.288**	0.275**	0.099	−0.173
	(0.110)	(0.128)	(0.140)	(0.070)	(0.280)
Drainage (under-ground or covered pucca)	0.011	0.152***	−0.059	−0.035	0.087
	(0.020)	(0.047)	(0.044)	(0.026)	(0.093)
Arranged garbage collection	−0.108	−0.226	−0.136	−0.047	−0.282
	(0.105)	(0.258)	(0.174)	(0.077)	(0.216)

Robust standard errors clustered at state levels in parentheses; ∗∗∗*p* < 0.01, ∗∗*p* < 0.05, ∗*p* < 0.1.

We further estimate the impact of different bundles of WASH practices. The results of these estimations, presented in Table 7, show that bundling at least six practices would bring the highest effects. In particular, the adoption of a bundle of six practices helps reduce the probability of having skin problems by 6.4%, fly and mosquito problems by 20.2%, stomach problems by 11.1%, and malaria problems by 13.2%. This confirms that WASH programs should aim at scaling practices in bundles rather than a single practice.

**Table 7:** Impacts of having different bundles of sanitation practices on health problems.

	Skin problems	Fly and mosquito problems	Stomach problems	Malaria problems	Other problems
	(1)	(2)	(3)	(4)	(6)
One practice	−0.074	−0.119	−0.129	0.175**	0.262
	(0.123)	(0.196)	(0.125)	(0.075)	(0.236)
Bundling two	0.001	−0.107	−0.069	0.125	−0.080
	(0.073)	(0.098)	(0.068)	(0.088)	(0.091)
Bundling three	−0.076**	−0.221*	−0.097	0.107	−0.174**
	(0.038)	(0.127)	(0.064)	(0.123)	(0.082)
Bundling four	−0.042	−0.119	0.006	0.045	−0.130*
	(0.037)	(0.120)	(0.069)	(0.109)	(0.070)
Bundling five	−0.027	−0.103*	−0.024	−0.139	−0.124
	(0.050)	(0.056)	(0.080)	(0.088)	(0.168)
Bundling six	−0.064*	−0.202***	−0.111***	−0.132***	−0.136
	(0.033)	(0.065)	(0.038)	(0.042)	(0.107)
Bundling more than six	−0.077**	−0.237***	−0.104***	−0.098**	−0.100
	(0.033)	(0.067)	(0.039)	(0.040)	(0.070)

Robust standard errors clustered at state levels in parentheses; ∗∗∗*p* < 0.01, ∗∗*p* < 0.05, ∗*p* < 0.1.

## 4 Discussion

Developing countries have made notable progress in expanding WASH practices, such as providing toilets and access to drinking water, in rural areas. However, these efforts often focus on individual practices, resulting in low adoption and limited overall impact. Moreover, rural populations are not homogeneous, and identifying the most disadvantaged groups is crucial to ensure inclusivity. Utilizing data from the 76th Round of the Indian National Sample Survey (NSS), Schedule 1.2, collected in 2018 for rural India, our study seeks to address three key questions: (i) what are associated with the adoption of various bundles of WASH practices? (ii) which population groups are more disadvantaged in adopting WASH practices? and (iii) what are the effects of adopted WASH practices on reducing related health issues? We focus on rural India as the problem of inadequate and unsafe WASH practices is particularly prevalent there (22–24). To investigate these questions, we employ a series of logit models for the first question, the Blinder-Oaxaca decomposition method for the second questions, and the heteroskedasticity-based instrumental variable (IV) approach for the third question. Additionally, we conduct a propensity score matching as a robustness check for our impact assessments.

Our analysis yields several important findings. First, we find that household wealth, as indicated by per capita consumption expenditure, and education levels of household leads are significant factors associated with the adoption of WASH practices. Second, our findings reveal that female-headed households and those belonging to scheduled castes and tribes face disadvantages in adopting WASH practices. Third, adopting a single WASH practice does not significantly alleviate sanitation-related health issues such as skin conditions and problems caused by flies, mosquitoes, stomach ailments, and malaria. However, implementing at least two WASH practices together significantly reduces these health problems. Notably, bundling six or more practices shows the most substantial impact in mitigating health issues.

Our findings that the adoption of WASH practices is significantly associated with several variables, including per capital consumption, age, gender, family size, family status (caste system), and education level of household heads are reasonable. The positive association between per capita consumption and the adoption of WASH practices underlines the importance of wealth status in the adoption. This is consistent with several previous studies, such as Gopalan & Rajan (25), Cameron et al. (26) and Biswas & Karmakar (27). These authors emphasize that limited purchasing power is a significant structural constraint restricting households' access to WASH practices. Cameron et al. (26) also posit that the impact of WASH interventions varies depending on a household's poverty status. For instance, a community-led total sanitation program that does not offer financial assistance to impoverished households might limit them to participate as they are not able to construct a toilet (28). Therefore, an increase in household purchasing power is likely to be associated with a higher level of WASH adoption. Our results further reveal that households headed by older individuals and those with larger family sizes are more inclined to adopt, including household-exclusive toilets, bathrooms, and drainage systems (underground or covered pucca). These findings are also corroborated in other studies such as Whittington et al. (29), Lipscomb and Schechter (30), and Acey et al. (31). Furthermore, our results indicate that education level is positively associated with the adoption of WASH practice, such as handwashing with soap before meals and after toilet use, as also demonstrated by Kariuki et al. (32) and Adukia (6).

Our results report the disparities observed among population groups regarding the adoption of WASH practices. In particular, households belonging to scheduled castes and tribes exhibit a lower propensity to embrace exclusive toilet and drainage (either underground or pucca-covered). This discrepancy may be attributed to several underlying factors, one prominent aspect being the capital-intensive nature of toilets and drainage services. Many of these tribal households face financial challenges, constraining their ability to invest in such sanitation facilities. These disparities in the adoption do not indicate poor implementation but rather stem from social structural constraints, including religious and caste-related factors. Our finding on the disparities in access to WASH practices is consistent with several previous studies [see a review by (33)]. Ezbakhe et al. (34) report that access to WAH practices of marginalized groups is constrainted. The results also highlight a significant gender gap in adopting single or bundled WASH practices. It is important to recognize that disadvantaged groups, such as scheduled castes and tribes, face distinct challenges in adopting sanitation services. Many of these households may need more financial means to invest in capital-intensive WASH facilities like toilets and drainage systems. This economic disparity, in turn, perpetuates unequal access to adequate WASH [see (35)]. Thus, addressing these structural constraints and promoting equitable access to sanitation is paramount. Regarding the gender gap, our research highlights the need for targeted interventions to empower female-headed households to adopt WASH practices. Equalizing access to resources and opportunities can significantly enhance these households' WASH practices. This, in turn, contributes to improved public health outcomes and overall well-being within communities.

Regarding the impacts of WASH adoption, our results show that adopting a single practice alone does not lead to a substantial improvement in health. This seems reasonable as previous evidence on the impact of a single practice is inconclusive. On the one hand, Jalan and Ravallion (36) and Kumar and Vollmer (37) report that access to piped water or improved sanitation in India reduces the prevalence of diarrhea among children by nearly 17%. Similarly, Augsburg and Rodríguez-Lesmes (38) find that higher latrine coverage is associated with increased child height in northern India. On the other hand, Wolf et al. (39, 40) conduct a systematic review of journal papers published between 1970 and 2013 to assess the impact of inadequate WASH practices on diarrheal disease in low- and middle-income countries. They find that on-site sanitation interventions without sewerage connectivity in rural areas fail to reduce diarrheal episodes. Cameron et al. (26) also explore the impact of a community-level sanitation intervention in Indonesia and conclude that such interventions can be counterproductive in villages with low social capital. Clasen et al. (10) and Patil et al. (11) evaluate the impact of a single sanitation service in rural India and find only a moderate impact of latrine construction on usage, with no significant effects on health outcomes. A notable insight from our research is that the cumulative impact of budling multiple practices is more pronounced. As households embrace more practices, the visible effect of reducing WASH-related health problems becomes more apparent. Prior literature in other disciplines suggests that complementarity exists among adopted practices, and analyzing a single practice's impact fails to capture these benefits. For example, Soh et al. (41) demonstrate that bundling several irrigation and nutrient best management practices enhances water quality and quantity more effectively than adopting a single management practice. In the sanitation literature, to our knowledge, only Duflo et al. (22) find that subsidizing private toilets (latrines) and bathing facilities equipped with tap water significantly increases toilet use and results in health gains. Our finding highlights the importance of promoting the adoption of bundled WASH practices. Results of the robustness check using PSM also show that adopting only one practice does not significantly reduce WASH-related health problems.^6^

Our study contributes to the literature by conducting a disaggregated analysis of the factors associated with adoption of single vs. multiple (bundled) WASH practices, exploring disparities in WASH adoption of marginalized population groups (female-headed households, scheduled castes and tribes) via the Blinder-Oaxaca decomposition method, and assessing the impacts of different bundles on reducing sanitation-related health problems in rural India. Unlike previous studies, our research examines numerous WASH practices, such as clean water provision, toilet use, handwashing, and solid and liquid waste disposal, assessing their combined impact on related communicable diseases. To our knowledge, this study is among the first to rigorously assess the impact of bundled WASH practices on health outcomes.

While our study offers valuable insights, it does have certain limitations. First, it accounts for a small number of variables at the household level, while the adoption might be influenced by other factors. For example, Novotný et al. (42) report that some psychosocial variables are found to be statistically significant correlates of toilet adoption. Second, it is unable to identify the heterogeneities in the impacts of adopting different WASH bundles. For example, Gopalan and Rajan (25) report that WASH interventions have a greater health impact on the poor compared to the better-off. Third, some previous studies, for example, Prakash et al. (13), use the concept of the Joint Monitoring Programme (JMP) sanitation ladder to classify sanitation from “no facility” to “safely managed service”. These ladders are useful for simplifying bundles, since there are numerous combinations of individual practices. Fourth, the cross-sectional nature of our data prevents us from examining intertemporal relationships and controlling for potential selection bias. Lastly, our analysis does not include urban regions. Addressing these issues should be a focus of future research efforts to enhance the robustness and comprehensiveness of the findings.

## 5 Conclusion

We find that limited purchasing power and low education level are significantly associated with low adoption of WASH practices. Female-headed households and those belonging to scheduled castes and tribes are disadvantaged in adopting WASH practices. Bundling several WASH practices is more effective in mitigating health problems compared to single-practice adoption. These findings suggest several important policy implications. First, there is a need to invest in enhancing the livelihoods and literacy levels of rural households to facilitate the adoption of WASH practices. Second, WASH programs and interventions should prioritize female-headed households and those belonging to scheduled castes and tribes, recognizing their particular challenges. Lastly, emphasizing the implementation of bundled WASH practices for impoverished households, those with lower educational attainment, female-headed households, and marginalized groups such as scheduled castes and tribes is essential for improving health outcomes and reducing the disease burden in rural India.

## Data Availability

The data analyzed in this study is subject to the following licenses/restrictions: We use statistical data provided by the Statistics Office of India Government. Researchers can contact this office for the data. Requests to access these datasets should be directed to thanh.nguyen@iuw.uni-hannover.de.

## Footnotes

^1^A detailed sampling methodology for this round can be found in NSS Report No. 584 (76/1.2/1), released by the Ministry of Statistics and Programme Implementation, Government of India (https://www.mospi.gov.in/unit-level-data-report-nss-76th-round-schedule-12-july-december-2018-drinking-water-sanitation). The description of the instrument can be found from https://www.mospi.gov.in/sites/default/files/NSS7612dws/NSS_76_sch_1.2.pdf.

^2^The results of these tests reported in Supplementary Tables S5–S7 confirm the validity of the IVs.

^3^Basic PSM approach in Supplementary Table S8. Results of our balancing tests (Supplementary Table S8D) show that most of the mean and median biases have been significantly reduced to less than 10% after matching.

^4^See Supplementary Tables S3A,B.

^5^See Supplementary Tables S4A,B.

^6^See Supplementary Tables S8A–C.
