# Supplementary material for: Improving the health of the rural population in India through bundling WASH practices
Source: Front Health Serv. 2025 Feb 19;5:1500504. doi: 10.3389/frhs.2025.1500504 (PMC11879964; doi:10.3389/frhs.2025.1500504)
Supplement: Supplementary file 1 [file Datasheet1.pdf]

Supplementary Information

Supplementary Table S1: Name, definition, and measurement of variables

Name	Measurement	Definition
**WASH practices**		
Drinking water (tap)	Dummy	If drinking water of the household comes from tab water within compound = 1; Otherwise = 0
Non-drinking water (tap)	Dummy	If non-drinking water of the household comes from tab water within compound = 1; Otherwise = 0
Treated drink- water	Dummy	If drinking water of the household is treated before consumption = 1; Otherwise: 0
Wash hand before meal with soap	Dummy	If the household does hand-washing with soap before meal = 1; Otherwise = 0
Toilet (exclusive use)	Dummy	If the household has a toilet which is for exclusive use of household = 1; Otherwise = 0
Bathroom (exclusive use)	Dummy	If the household has a bathroom which is for exclusive use of household = 1; Otherwise = 0
Drainage (underground or covered pucca)	Dummy	If the household has a drainage which is underground or covered pucca = 1; Otherwise = 0
Arranged garbage collection	Dummy	If the household has arranged garbage collection = 1; Otherwise = 0
**WASH related health problems**		
Skin problems	Dummy	If the household had skin diseases in the last 365 days = 1; Otherwise = 0
Fly and mosquito problems	Dummy	If the household lived in area with a lot of flies/mosquitoes in the last 365 days = 1; Otherwise = 0
Stomach problems	Dummy	If the household faced stomach problems like diarrhea/dysentery/cholera in the last 365 days = 1; Otherwise = 0
Malaria problems	Dummy	If the household faced Malaria/dengue/chikungunya/encephalitis in the last 365 days = 1; Otherwise = 0
Other problems	Dummy	If the household faced other WASH related diseases in the last 365 days = 1; Otherwise = 0
**Household's characteristics**		
Monthly expenditure per capita (INR)	Indian Rupee (INR)	Monthly household's expenditure per capita
Female head	Dummy	If the household is female-headed = 1; Otherwise = 0
Age head (years)	Years of age	Age of the household head
Household size	Persons	Number of household members
No. of males	Persons	Number of male members in the household
No. of children	Children	Number of children who are younger than 14 years old in the household
No. of elders	Persons	Number of elders who are older than 60 years old in the household
Illiterate head	Dummy	If the household head is illiterate = 1; Otherwise = 0
High school head	Dummy	If the highest education level of the household head is high school = 1; Otherwise = 0
Diploma head	Dummy	If the highest education level of the household head is diploma certificate course = 1; Otherwise = 0
University and above head	Dummy	If the highest education level of the household head is university degree or higher = 1; Otherwise = 0
Scheduled tribes	Dummy	If the household belongs to the scheduled tribes (ST) = 1; Otherwise = 0
Scheduled castes	Dummy	If the household belongs to the scheduled castes (SC) = 1; Otherwise = 0
Hindu	Dummy	If the religion of the household is Hindu = 1; Otherwise = 0
Islam	Dummy	If the religion of the household is Islam = 1; Otherwise = 0
Christianity	Dummy	If the religion of the household is Christianity = 1; Otherwise = 0
Sikhism	Dummy	If the religion of the household is Sikhism = 1; Otherwise = 0
Buddhism	Dummy	If the religion of the household is Buddhism = 1; Otherwise = 0

Supplementary Table S2: Variance Inflator Factor (VIF) values of independent variables in the adoption models

Variable	VIF	1/VIF
Monthly expenditure per capita	1.74	0.57
Female head	1.23	0.81
Age head	2.02	0.49
HH size	3.51	0.29
No. of males	4.25	0.24
No. of children	2.34	0.43
No. of elders	1.98	0.50
Illiterate head	1.32	0.76
High school head	1.08	0.92
Diploma head	1.02	0.98
Uni and above head	1.09	0.92
Scheduled castes	1.30	0.77
Scheduled tribes	1.16	0.86
Christianity	3.60	0.28
Sikhism	4.15	0.24
Buddhism	1.47	0.68
State dummy (Base = Jammu and Kashmir)		
Himachal Pradesh	1.97	0.51
Punjab	4.72	0.21
Chandigarh	1.01	0.99
Uttarakhand	1.94	0.52
Haryana	3.01	0.33
Delhi	1.07	0.94
Rajasthan	5.73	0.17
Uttar Pradesh	12.03	0.08
Bihar	8.51	0.12
Sikkim	1.09	0.92
Arunachal Pradesh	1.17	0.86
Nagaland	1.26	0.79
Manipur	1.28	0.78
Mizoram	1.11	0.90
Tripura	1.38	0.72
Meghalaya	1.45	0.69
Assam	4.03	0.25
West Bengal	6.23	0.16
Jharkhand	2.98	0.34
Odisha	4.77	0.21
Chhattisgarh	3.16	0.32
Madhya Pradesh	5.88	0.17
Gujarat	4.25	0.24
Daman & Diu	1.01	0.99
D and N Haveli	1.02	0.98
Maharashtra	7.54	0.13
Andhra Pradesh	6.02	0.17
Karnataka	5.02	0.20
Goa	1.08	0.92
Lakshadweep	1.00	1.00
Kerala	3.86	0.26
Tamil Nadu	5.28	0.19
Puducherry	1.06	0.94
A and N Islands	1.02	0.98
Telangana	3.83	0.26
**Mean VIF**	**3.27**	

Supplementary Table S3A: Counter factual analysis of adopting only one practice between female- and male-headed households

	Probabilities of adopting only one specific sanitation practice						
	Drinking water (tap)	Treated drink-water	Wash hand before meal with soap	Toilet (exclusive use)	Bathroom (exclusive use)	Drainage (underground or covered pucca)	Arranged garbage collection
	(1)	(2)	(3)	(4)	(5)	(6)	(7)
**Differential**							
Male-headed	0.011***	0.142***	0.082***	0.480***	0.093***	0.077***	0.107***
	(0.001)	(0.014)	(0.002)	(0.017)	(0.004)	(0.006)	(0.005)
Female-headed	0.010***	0.121***	0.078***	0.423***	0.082***	0.086***	0.133***
	(0.001)	(0.012)	(0.003)	(0.025)	(0.004)	(0.008)	(0.008)
Difference	0.001	0.021***	0.004***	0.057***	0.012***	-0.009***	-0.026***
	(0.001)	(0.006)	(0.001)	(0.009)	(0.002)	(0.002)	(0.004)
**Decomposition**							
Endowments [a]	-0.005***	0.002	0.019***	0.046***	0.014***	0.025***	-0.007**
	(0.001)	(0.005)	(0.001)	(0.009)	(0.002)	(0.002)	(0.004)
Coefficients [b]	0.006***	0.018***	-0.014***	0.010***	-0.004***	-0.030***	-0.018***
	(0.000)	(0.002)	(0.001)	(0.000)	(0.001)	(0.002)	(0.002)
Interaction [c]	-0.000*	0.001	-0.001	0.001*	0.002	-0.004***	-0.000
	(0.000)	(0.001)	(0.000)	(0.000)	(0.001)	(0.001)	(0.001)
Number of observations	8007	9362	8710	15602	8858	8600	8921

Robust standard errors clustered at state levels; *** p<0.01, ** p<0.05, * p<0.1; [a]: The change of the probabilities of adopting only one practice in female-headed households if they had the same characteristics as male-headed households; [b]: The change of the probabilities of adopting only one practice in female-headed households when applying the coefficients of male-headed households to female-headed households; [c]: The interaction term accounting for the fact that differences in endowments and coefficients exist simultaneously between (a) and (b).

Supplementary Table S3B: Counter factual analysis of adopting one practice along with at least another practice between female- and male-headed households

	Probabilities of adopting a specific sanitation practice along with at least another practice							
	Drinking water (tap)	Non-drinking water (tap)	Treated drink-water	Wash hand before meal with soap	Toilet (exclusive use)	Bathroom (exclusive use)	Drainage (underground or covered pucca)	Arranged garbage collection
	(1)	(2)	(3)	(4)	(5)	(6)	(7)	(8)
**Differential**								
Male-headed	0.512***	0.490***	0.629***	0.624***	0.801***	0.772***	0.575***	0.546***
	(0.045)	(0.046)	(0.037)	(0.035)	(0.018)	(0.022)	(0.037)	(0.042)
Female-headed	0.486***	0.460***	0.627***	0.597***	0.783***	0.746***	0.561***	0.561***
	(0.047)	(0.050)	(0.050)	(0.043)	(0.025)	(0.031)	(0.050)	(0.048)
Difference	0.025	0.030	0.002	0.027	0.018	0.026*	0.014	-0.016
	(0.019)	(0.018)	(0.025)	(0.019)	(0.013)	(0.015)	(0.024)	(0.020)
**Decomposition**								
Endowments [a]	0.064***	0.077***	0.056**	0.074***	0.041***	0.039**	0.074***	0.056***
	(0.019)	(0.018)	(0.026)	(0.019)	(0.013)	(0.016)	(0.024)	(0.020)
Coefficients [b]	-0.042***	-0.046***	-0.062***	-0.052***	-0.037***	-0.022***	-0.070***	-0.077***
	(0.004)	(0.004)	(0.004)	(0.003)	(0.004)	(0.004)	(0.006)	(0.004)
Interaction [c]	0.003	-0.001	0.008*	0.005	0.014***	0.009**	0.010**	0.005
	(0.006)	(0.004)	(0.005)	(0.004)	(0.004)	(0.004)	(0.005)	(0.005)
Number of observations	18240	17685	28227	24791	47984	41133	19786	19913

Robust standard errors clustered at state levels; *** p<0.01, ** p<0.05, * p<0.1; [a]: The change of probabilities of adopting only one practice along with at least another practice in female-headed households if they had the same characteristics as male-headed households; [b]: The change of the probabilities of adopting only one practice along with at least another practice in female-headed households when applying the coefficients of male-headed households to female-headed households; [c]: The interaction term accounting for the fact that differences in endowments and coefficients exist simultaneously between (a) and (b).

Supplementary Table S4A: Counter factual analysis of adopting only one specific practice between households in the scheduled castes (SC) or scheduled tribes (ST) and the rest of households

	Probabilities of adopting only one specific sanitation practice						
	Drinking water (tap)	Treated drink-water	Wash hand before meal with soap	Toilet (exclusive use)	Bathroom (exclusive use)	Drainage (underground or covered pucca)	Arranged garbage collection
	(1)	(2)	(3)	(4)	(5)	(6)	(7)
**Differential**							
Other households	0.011***	0.116***	0.093***	0.504***	0.106***	0.100***	0.114***
	(0.001)	(0.005)	(0.002)	(0.023)	(0.005)	(0.004)	(0.006)
ST or SC households	0.010***	0.165***	0.068***	0.433***	0.075***	0.052***	0.108***
	(0.001)	(0.022)	(0.003)	(0.013)	(0.005)	(0.007)	(0.009)
Difference	0.002	-0.049***	0.025***	0.071***	0.032***	0.049***	0.006
	(0.001)	(0.019)	(0.002)	(0.012)	(0.004)	(0.005)	(0.010)
**Decomposition**							
Endowments [a]	0.004***	0.101***	0.000	0.073***	0.009**	0.021***	0.031***
	(0.001)	(0.019)	(0.002)	(0.012)	(0.004)	(0.006)	(0.011)
Coefficients [b]	0.002	-0.052***	0.014***	-0.015***	0.026***	0.042***	-0.003
	(0.001)	(0.019)	(0.002)	(0.004)	(0.002)	(0.005)	(0.008)
Interaction [c]	-0.004***	-0.099***	0.011***	0.013***	-0.003	-0.015**	-0.023***
	(0.001)	(0.019)	(0.002)	(0.004)	(0.002)	(0.006)	(0.008)
Number of observations	8007	9362	8710	15602	8858	8600	8921

Robust standard errors clustered at state levels; *** p<0.01, ** p<0.05, * p<0.1; [a]: The change of the probabilities of adopting one specific sanitation practice in the households in ST and SC social groups if they had the same characteristics as households in the other social groups; [b]: The change of the probabilities of adopting one specific sanitation practice in the households in ST and SC social groups when applying the coefficients of households in in the other social groups to the households in ST and SC social group; [c]: The interaction term accounting for the fact that differences in endowments and coefficients exist simultaneously between (a) and (b).

Supplementary Table S4B: Counter factual analysis of adopting one specific practice along with at least another practice between households in the scheduled castes (SC) or scheduled tribes (ST) and the rest of households

	Probabilities of adopting a specific sanitation practice along with at least another practice							
	Drinking water (tap)	Non-drinking water (tap)	Treated drink-water	Wash hand before meal with soap	Toilet (exclusive use)	Bathroom (exclusive use)	Drainage (underground or covered pucca)	Arranged garbage collection
	(1)	(2)	(3)	(4)	(5)	(6)	(7)	(8)
**Differential**								
Other households	0.590*** (0.041)	0.566*** (0.044)	0.688*** (0.034)	0.691*** (0.032)	0.842*** (0.015)	0.821*** (0.018)	0.666*** (0.036)	0.616*** (0.039)
ST or SC households	0.364*** (0.045)	0.348*** (0.046)	0.526*** (0.042)	0.488*** (0.040)	0.705*** (0.027)	0.655*** (0.033)	0.376*** (0.036)	0.431*** (0.044)
Difference	0.226*** (0.020)	0.218*** (0.021)	0.162*** (0.025)	0.202*** (0.018)	0.138*** (0.016)	0.166*** (0.019)	0.290*** (0.023)	0.185*** (0.018)
**Decomposition**								
Endowments [a]	0.144*** (0.018)	0.147*** (0.020)	0.055** (0.027)	0.129*** (0.016)	0.079*** (0.014)	0.108*** (0.016)	0.190*** (0.025)	0.119*** (0.018)
Coefficients [b]	0.094*** (0.009)	0.081*** (0.009)	0.048*** (0.016)	0.079*** (0.012)	0.072*** (0.011)	0.087*** (0.013)	0.139*** (0.012)	0.076*** (0.006)
Interaction [c]	-0.012 (0.010)	-0.010 (0.010)	0.059*** (0.016)	-0.005 (0.008)	-0.013* (0.007)	-0.030*** (0.008)	-0.039*** (0.013)	-0.010 (0.006)
Number of observations	18240	17685	28227	24791	47984	41133	19786	19913

Robust standard errors clustered at state levels; *** p<0.01, ** p<0.05, * p<0.1; [a]: The change of the probabilities of adopting one specific practice along with at least another practice in the households in ST and SC social groups if they had the same characteristics as households in the other social groups; [b]: The change of the probabilities of adopting one specific practice along with at least another practice in the households in ST and SC social groups when applying the coefficients of households in in the other social groups to the households in ST and SC social group; [c]: The interaction term accounting for the fact that differences in endowments and coefficients exist simultaneously between (a) and (b).

Supplementary Table S5A: Impacts of adopting only drinking water (tap) on health problems (IV regressions)

	Skin problems	Fly and mosquito problems	Stomach problems	Malaria problems	Other problems
	(1)	(2)	(3)	(4)	(5)
Drinking water (tap)	-0.050	0.031	-0.067	-0.061	-0.187*
	(0.035)	(0.111)	(0.090)	(0.053)	(0.095)
Monthly expenditure per capita (ln)	0.013	-0.037	-0.029	-0.004	-0.069
	(0.030)	(0.040)	(0.025)	(0.039)	(0.053)
Female head	0.011	-0.033	-0.039*	-0.017	-0.068***
	(0.019)	(0.035)	(0.022)	(0.011)	(0.016)
Age head	-0.000	-0.002**	-0.000	0.000	0.000
	(0.000)	(0.001)	(0.000)	(0.000)	(0.001)
Household size	0.011**	0.001	0.022***	0.015**	0.007
	(0.005)	(0.007)	(0.007)	(0.006)	(0.011)
No. of males	-0.004	0.012	-0.000	-0.006	-0.002
	(0.007)	(0.011)	(0.013)	(0.004)	(0.011)
No. of children	-0.009***	-0.012	-0.038***	-0.003	-0.005
	(0.004)	(0.010)	(0.010)	(0.012)	(0.012)
No. of elders	-0.001	-0.010	-0.023	-0.007	0.012*
	(0.004)	(0.011)	(0.016)	(0.009)	(0.006)
Illiterate head	0.002	0.052***	-0.002	-0.022*	-0.036
	(0.013)	(0.017)	(0.017)	(0.013)	(0.037)
High school head	-0.026**	-0.001	-0.014	0.030*	-0.014
	(0.013)	(0.024)	(0.021)	(0.017)	(0.018)
Diploma head	0.064**	-0.063	0.014	-0.097**	-0.071
	(0.032)	(0.197)	(0.072)	(0.046)	(0.088)
Uni and above head	-0.022*	0.093*	-0.014	0.062	-0.006
	(0.013)	(0.053)	(0.030)	(0.048)	(0.035)
Scheduled tribes	-0.037**	-0.174***	-0.002	0.002	0.098
	(0.016)	(0.033)	(0.019)	(0.025)	(0.060)
Scheduled castes	-0.003	-0.045*	0.009	-0.015	-0.011
	(0.005)	(0.026)	(0.015)	(0.014)	(0.017)
Hindu	0.012	-0.079	-0.021	-0.006	0.067
	(0.019)	(0.068)	(0.048)	(0.055)	(0.046)
Islam	0.047	-0.084	-0.006	-0.068	0.065
	(0.041)	(0.073)	(0.055)	(0.058)	(0.041)
Christianity	-0.027	-0.155	-0.006	0.100	-0.080
	(0.019)	(0.137)	(0.114)	(0.068)	(0.095)
Sikhism	0.066	0.022	0.072	-0.063	0.087
	(0.083)	(0.090)	(0.118)	(0.063)	(0.100)
Buddhism	-0.069***	-0.500***	-0.090	-0.153***	-0.347***
	(0.022)	(0.091)	(0.080)	(0.046)	(0.054)
constant	-0.017	1.024***	0.406**	0.149	0.815**
	(0.223)	(0.328)	(0.158)	(0.317)	(0.409)
Number of observations	8030	8030	8030	8030	8030
R^2	0.009	0.022	0.015	0.012	0.019
Adjusted R^2	0.007	0.020	0.013	0.010	0.017
Under-identification	0.356	0.356	0.356	0.356	0.356
Over-identification	N/A	N/A	N/A	N/A	N/A
Weak identification	196.399	196.399	196.399	196.399	196.399

Robust standard errors clustered at state levels; N/A: Not applicable; *** $p<0.01$, ** $p<0.05$, * $p<0.1$.

Supplementary Table S5B: Impacts of adopting only non-drinking water (tap) on health problems (IV regressions)

	Skin problems	Fly and mosquito problems	Stomach problems	Malaria problems	Other problems
	(1)	(2)	(3)	(4)	(5)
Non-drinking water (tap)	-0.099***	0.163	-0.004	0.043	-0.129
	(0.027)	(0.101)	(0.053)	(0.080)	(0.083)
Monthly expenditure per capita (ln)	0.014	-0.038	-0.028	-0.004	-0.073
	(0.030)	(0.041)	(0.024)	(0.039)	(0.054)
Female head	0.010	-0.038	-0.040*	-0.017	-0.069***
	(0.020)	(0.034)	(0.022)	(0.010)	(0.015)
Age head	-0.000	-0.002**	-0.000	0.000	0.000
	(0.000)	(0.001)	(0.000)	(0.000)	(0.001)
Household size	0.012**	0.000	0.023***	0.015**	0.006
	(0.005)	(0.008)	(0.007)	(0.006)	(0.011)
No. of males	-0.004	0.014	-0.001	-0.005	-0.001
	(0.007)	(0.011)	(0.013)	(0.004)	(0.011)
No. of children	-0.009***	-0.014	-0.039***	-0.003	-0.005
	(0.004)	(0.010)	(0.010)	(0.012)	(0.012)
No. of elders	0.000	-0.014	-0.023	-0.007	0.010*
	(0.004)	(0.012)	(0.015)	(0.009)	(0.006)
Illiterate head	0.003	0.051***	-0.003	-0.022*	-0.036
	(0.013)	(0.017)	(0.017)	(0.013)	(0.038)
High school head	-0.025*	-0.003	-0.018	0.032*	-0.014
	(0.013)	(0.023)	(0.021)	(0.017)	(0.018)
Diploma head	0.065**	-0.057	0.014	-0.098**	-0.070
	(0.032)	(0.199)	(0.073)	(0.047)	(0.089)
Uni and above head	-0.022*	0.078	-0.016	0.072	-0.006
	(0.013)	(0.053)	(0.031)	(0.051)	(0.036)
Scheduled tribes	-0.037**	-0.174***	-0.003	0.001	0.098
	(0.016)	(0.033)	(0.020)	(0.025)	(0.061)
Scheduled castes	-0.004	-0.045*	0.010	-0.017	-0.013
	(0.004)	(0.026)	(0.015)	(0.014)	(0.017)
Hindu	0.012	-0.076	-0.023	-0.007	0.064
	(0.020)	(0.069)	(0.049)	(0.057)	(0.047)
Islam	0.047	-0.084	-0.008	-0.070	0.060
	(0.041)	(0.074)	(0.056)	(0.060)	(0.042)
Christianity	-0.026	-0.147	-0.004	0.084	-0.083
	(0.019)	(0.138)	(0.115)	(0.068)	(0.096)
Sikhism	0.065	0.025	0.069	-0.064	0.087
	(0.083)	(0.089)	(0.118)	(0.064)	(0.100)
Buddhism	-0.069***	-0.499***	-0.092	-0.155***	-0.349***
	(0.022)	(0.092)	(0.081)	(0.047)	(0.054)
constant	-0.024	1.025***	0.399***	0.152	0.849**
	(0.222)	(0.339)	(0.152)	(0.318)	(0.418)
Number of observations	8002	8002	8002	8002	8002
R^2	0.009	0.023	0.014	0.012	0.017
Adjusted R^2	0.007	0.021	0.012	0.009	0.015
Under-identification	0.086	0.086	0.086	0.086	0.086
Over-identification	N/A	N/A	N/A	N/A	N/A
Weak identification	7877.032	7877.032	7877.032	7877.032	7877.032

Robust standard errors clustered at state levels; N/A: Not applicable; *** p<0.01, ** p<0.05, * p<0.1.

Supplementary Table S5C: Impacts of adopting only treated drinking water on health problems (IV regressions)

	Skin problems	Fly and mosquito problems	Stomach problems	Malaria problems	Other problems
	(1)	(2)	(3)	(4)	(5)
Treated drinking water	-0.064	-0.093	0.156	-0.237	-0.037
	(0.087)	(0.193)	(0.140)	(0.189)	(0.099)
Monthly expenditure per capita (ln)	0.027	-0.057	-0.059	0.026	-0.089
	(0.028)	(0.036)	(0.039)	(0.054)	(0.060)
Female head	0.013	-0.028	-0.033	-0.021*	-0.061***
	(0.017)	(0.033)	(0.022)	(0.012)	(0.015)
Age head	-0.000	-0.001*	-0.000	0.000	0.000
	(0.000)	(0.001)	(0.000)	(0.000)	(0.001)
Household size	0.012***	-0.002	0.020***	0.015**	0.005
	(0.005)	(0.007)	(0.008)	(0.007)	(0.011)
No. of males	-0.003	0.012	-0.002	-0.005	-0.004
	(0.007)	(0.011)	(0.013)	(0.006)	(0.010)
No. of children	-0.009***	-0.012	-0.033***	-0.002	-0.002
	(0.003)	(0.010)	(0.011)	(0.013)	(0.012)
No. of elders	-0.001	-0.014	-0.024*	-0.007	0.015**
	(0.004)	(0.011)	(0.014)	(0.008)	(0.007)
Illiterate head	0.003	0.045***	-0.002	-0.022**	-0.028
	(0.011)	(0.016)	(0.015)	(0.011)	(0.034)
High school head	-0.030**	-0.000	0.003	-0.000	-0.000
	(0.014)	(0.021)	(0.026)	(0.027)	(0.022)
Diploma head	0.057*	-0.015	0.019	-0.079	-0.071
	(0.032)	(0.200)	(0.075)	(0.062)	(0.089)
Uni and above head	-0.022**	0.053	-0.004	0.054	0.020
	(0.010)	(0.077)	(0.035)	(0.046)	(0.039)
Scheduled tribes	-0.021	-0.122***	-0.020	0.052	0.081
	(0.018)	(0.038)	(0.027)	(0.048)	(0.054)
Scheduled castes	-0.005	-0.032	0.010	-0.021	-0.017
	(0.006)	(0.027)	(0.015)	(0.016)	(0.015)
Hindu	-0.005	-0.107***	-0.045	-0.060	0.037
	(0.028)	(0.041)	(0.038)	(0.094)	(0.044)
Islam	0.021	-0.102*	-0.023	-0.137	0.033
	(0.045)	(0.059)	(0.042)	(0.100)	(0.039)
Christianity	-0.050**	-0.149	0.006	0.028	-0.034
	(0.026)	(0.112)	(0.107)	(0.097)	(0.086)
Sikhism	0.036	-0.006	0.074	-0.165	0.065
	(0.079)	(0.066)	(0.123)	(0.105)	(0.100)
Buddhism	0.025	-0.341***	-0.064	-0.013	-0.268***
	(0.040)	(0.105)	(0.091)	(0.138)	(0.054)
constant	-0.106	1.177***	0.635**	0.010	1.000**
	(0.203)	(0.290)	(0.266)	(0.424)	(0.451)
Number of observations	9362	9362	9362	9362	9362
R^2	0.006	0.024	-0.004	-0.044	0.016
Adjusted R^2	0.004	0.022	-0.006	-0.046	0.014
Under-identification	0.293	0.293	0.293	0.293	0.293
Over-identification	0.723	0.171	0.400	0.888	0.407
Weak identification	15.381	15.381	15.381	15.381	15.381

Robust standard errors clustered at state levels; *** $p<0.01$, ** $p<0.05$, * $p<0.1$.

Supplementary Table S5D: Impacts of adopting only wash-hand-before-meal-with-soap on health problems (IV regressions)

	Skin problems	Fly and mosquito problems	Stomach problems	Malaria problems	Other problems
	(1)	(2)	(3)	(4)	(5)
Wash hand before meal with soap	0.097	0.003	-0.085	-0.017	-0.250
	(0.084)	(0.173)	(0.127)	(0.043)	(0.237)
Monthly expenditure per capita (ln)	0.006	-0.035	-0.018	-0.002	-0.052
	(0.030)	(0.038)	(0.027)	(0.042)	(0.050)
Female head	0.008	-0.037	-0.038*	-0.012	-0.060***
	(0.020)	(0.030)	(0.023)	(0.010)	(0.017)
Age head	-0.000	-0.001**	0.000	0.000	-0.000
	(0.000)	(0.001)	(0.000)	(0.000)	(0.001)
Household size	0.012***	-0.000	0.021***	0.016***	0.006
	(0.004)	(0.007)	(0.006)	(0.006)	(0.010)
No. of males	-0.006	0.013	0.002	-0.005*	0.003
	(0.007)	(0.012)	(0.012)	(0.003)	(0.010)
No. of children	-0.008**	-0.009	-0.037***	-0.001	-0.009
	(0.003)	(0.011)	(0.009)	(0.010)	(0.011)
No. of elders	0.000	-0.011	-0.024	-0.005	0.011**
	(0.004)	(0.011)	(0.015)	(0.007)	(0.005)
Illiterate head	-0.000	0.049***	-0.004	-0.025**	-0.035
	(0.013)	(0.016)	(0.017)	(0.012)	(0.037)
High school head	-0.022	0.004	-0.004	0.021	-0.029
	(0.016)	(0.019)	(0.021)	(0.016)	(0.020)
Diploma head	0.068**	-0.039	0.041	-0.101**	-0.057
	(0.030)	(0.190)	(0.072)	(0.046)	(0.086)
Uni and above head	-0.032***	0.098**	-0.016	0.036	-0.021
	(0.011)	(0.045)	(0.029)	(0.039)	(0.036)
Scheduled tribes	-0.033**	-0.169***	-0.006	0.001	0.088
	(0.015)	(0.033)	(0.018)	(0.025)	(0.061)
Scheduled castes	-0.001	-0.040*	0.011	-0.009	-0.012
	(0.005)	(0.023)	(0.015)	(0.014)	(0.018)
Hindu	0.021	-0.081	-0.017	0.008	0.045
	(0.017)	(0.061)	(0.046)	(0.057)	(0.053)
Islam	0.051	-0.103	-0.008	-0.046	0.049
	(0.034)	(0.066)	(0.050)	(0.065)	(0.049)
Christianity	-0.017	-0.168	-0.006	0.090	-0.115
	(0.019)	(0.130)	(0.112)	(0.069)	(0.103)
Sikhism	0.109	-0.068	0.068	-0.053	0.053
	(0.069)	(0.107)	(0.102)	(0.059)	(0.097)
Buddhism	-0.031*	-0.489***	-0.094	-0.139***	-0.374***
	(0.018)	(0.092)	(0.081)	(0.049)	(0.054)
constant	0.004	0.998***	0.314*	0.111	0.739*
	(0.222)	(0.301)	(0.169)	(0.334)	(0.384)
Number of observations	8710	8710	8710	8710	8710
R^2	-0.003	0.021	0.013	0.011	0.007
Adjusted R^2	-0.005	0.019	0.011	0.009	0.005
Under-identification	0.369	0.369	0.369	0.369	0.369
Over-identification	N/A	N/A	N/A	N/A	N/A
Weak identification	40.823	40.823	40.823	40.823	40.823

Robust standard errors clustered at state levels; N/A: Not applicable; *** $p<0.01$, ** $p<0.05$, * $p<0.1$.

Supplementary Table S5E: Impacts of adopting only toilet (exclusive use) on health problems (IV regressions)

	Skin problems	Fly and mosquito problems	Stomach problems	Malaria problems	Other problems
	(1)	(2)	(3)	(4)	(5)
Toilet (exclusive use)	0.164	0.154	0.281	0.184	0.243
	(0.223)	(0.277)	(0.279)	(0.308)	(0.285)
Monthly expenditure per capita (ln)	-0.015	-0.064	-0.083	-0.044	-0.104
	(0.033)	(0.067)	(0.062)	(0.057)	(0.092)
Female head	0.006	-0.036	-0.022	0.006	-0.045***
	(0.021)	(0.032)	(0.021)	(0.008)	(0.012)
Age head	-0.001	-0.001	-0.001*	-0.001	-0.001
	(0.001)	(0.001)	(0.001)	(0.001)	(0.001)
Household size	0.011***	0.003	0.015***	0.013***	0.007
	(0.003)	(0.005)	(0.004)	(0.005)	(0.009)
No. of males	0.003	0.014*	0.013	0.004	0.008
	(0.005)	(0.008)	(0.010)	(0.009)	(0.008)
No. of children	-0.016**	-0.017*	-0.039***	-0.009	-0.016
	(0.008)	(0.009)	(0.013)	(0.016)	(0.014)
No. of elders	-0.004	-0.007	-0.012	0.002	-0.001
	(0.004)	(0.008)	(0.012)	(0.006)	(0.005)
Illiterate head	0.017	0.070**	0.033	0.014	0.012
	(0.024)	(0.036)	(0.038)	(0.043)	(0.050)
High school head	-0.030**	0.024	-0.040*	-0.015	-0.015
	(0.013)	(0.036)	(0.024)	(0.013)	(0.015)
Diploma head	0.065	-0.012	0.020	-0.003	0.014
	(0.055)	(0.103)	(0.085)	(0.042)	(0.080)
Uni and above head	-0.045*	0.009	-0.043	0.009	-0.024
	(0.024)	(0.051)	(0.057)	(0.033)	(0.041)
Scheduled tribes	-0.043**	-0.158***	-0.023	0.022	0.050
	(0.020)	(0.045)	(0.027)	(0.043)	(0.058)
Scheduled castes	-0.003	-0.033	0.004	-0.010	-0.009
	(0.004)	(0.025)	(0.018)	(0.021)	(0.019)
Hindu	-0.025	-0.086	-0.098**	-0.059	-0.001
	(0.038)	(0.058)	(0.041)	(0.044)	(0.072)
Islam	-0.050	-0.132	-0.170*	-0.164**	-0.018
	(0.087)	(0.090)	(0.095)	(0.082)	(0.123)
Christianity	-0.071	-0.138	-0.091	-0.021	-0.097
	(0.052)	(0.118)	(0.114)	(0.033)	(0.124)
Sikhism	0.002	-0.020	0.142	-0.036	-0.032
	(0.064)	(0.119)	(0.089)	(0.086)	(0.078)
Buddhism	-0.125*	-0.307***	-0.116	-0.092	-0.316***
	(0.068)	(0.112)	(0.082)	(0.076)	(0.081)
constant	0.153	1.112**	0.764**	0.403	1.037*
	(0.192)	(0.465)	(0.373)	(0.370)	(0.626)
Number of observations	15602	15602	15602	15602	15602
R^2	-0.066	-0.014	-0.118	-0.073	-0.047
Adjusted R^2	-0.067	-0.015	-0.119	-0.074	-0.049
Under-identification	0.716	0.716	0.716	0.716	0.716
Over-identification	0.684	0.663	0.562	0.272	0.189
Weak identification	1.792	1.792	1.792	1.792	1.792

Robust standard errors clustered at state levels; *** p<0.01, ** p<0.05, * p<0.1.

Supplementary Table S5F: Impacts of adopting only bathroom (exclusive use) on health problems (IV regressions)

	Skin problems	Fly and mosquito problems	Stomach problems	Malaria problems	Other problems
	(1)	(2)	(3)	(4)	(5)
Bathroom (exclusive use)	-0.080	-0.288**	0.275**	0.099	-0.173
	(0.110)	(0.128)	(0.140)	(0.070)	(0.280)
Monthly expenditure per capita (ln)	0.013	-0.008	-0.079**	-0.018	-0.068
	(0.041)	(0.035)	(0.034)	(0.042)	(0.056)
Female head	0.014	-0.032	-0.039*	-0.009	-0.068***
	(0.019)	(0.031)	(0.023)	(0.010)	(0.018)
Age head	-0.000	-0.002**	-0.000	-0.000	0.000
	(0.000)	(0.001)	(0.000)	(0.000)	(0.001)
Household size	0.011**	0.003	0.016**	0.014***	0.005
	(0.005)	(0.007)	(0.006)	(0.005)	(0.010)
No. of males	-0.001	0.013	0.005	-0.003	0.003
	(0.005)	(0.010)	(0.012)	(0.005)	(0.009)
No. of children	-0.010***	-0.015*	-0.035***	-0.003	-0.006
	(0.003)	(0.009)	(0.010)	(0.011)	(0.011)
No. of elders	-0.003	-0.010	-0.025*	-0.004	0.010**
	(0.004)	(0.010)	(0.013)	(0.010)	(0.005)
Illiterate head	0.002	0.054***	0.006	-0.019*	-0.033
	(0.012)	(0.018)	(0.017)	(0.011)	(0.037)
High school head	-0.025**	-0.010	-0.003	0.028	-0.030*
	(0.011)	(0.020)	(0.020)	(0.022)	(0.018)
Diploma head	0.051*	-0.105	0.059	-0.044	-0.091
	(0.029)	(0.181)	(0.077)	(0.063)	(0.081)
Uni and above head	-0.027**	0.115**	-0.016	0.046	0.052
	(0.012)	(0.047)	(0.021)	(0.042)	(0.052)
Scheduled tribes	-0.026	-0.171***	0.011	0.007	0.087
	(0.019)	(0.027)	(0.023)	(0.025)	(0.060)
Scheduled castes	-0.004	-0.041	0.014	-0.014	-0.012
	(0.005)	(0.025)	(0.017)	(0.013)	(0.018)
Hindu	0.013	-0.073	-0.010	0.002	0.065
	(0.018)	(0.060)	(0.045)	(0.052)	(0.046)
Islam	0.045	-0.081	0.015	-0.056	0.059
	(0.040)	(0.068)	(0.044)	(0.054)	(0.039)
Christianity	-0.033*	-0.146	-0.012	0.106*	-0.078
	(0.017)	(0.119)	(0.118)	(0.060)	(0.090)
Sikhism	0.065	-0.065	-0.066	-0.105***	0.206*
	(0.054)	(0.078)	(0.083)	(0.038)	(0.114)
Buddhism	-0.055**	-0.130	-0.119	-0.080	-0.298***
	(0.027)	(0.158)	(0.088)	(0.053)	(0.080)
constant	-0.019	0.814***	0.736***	0.235	0.820*
	(0.301)	(0.294)	(0.235)	(0.325)	(0.421)
Number of observations	8858	8858	8858	8858	8858
R^2	0.005	0.009	-0.022	0.004	0.017
Adjusted R^2	0.003	0.007	-0.024	0.002	0.015
Under-identification	0.223	0.223	0.223	0.223	0.223
Over-identification	N/A	N/A	N/A	N/A	N/A
Weak identification	27.171	27.171	27.171	27.171	27.171

Robust standard errors clustered at state levels; N/A: Not applicable; *** p<0.01, ** p<0.05, * p<0.1.

Supplementary Table S5G: Impacts of adopting only drainage (underground or covered pucca) on health problems (IV regressions)

	Skin problems	Fly and mosquito problems	Stomach problems	Malaria problems	Other problems
	(1)	(2)	(3)	(4)	(5)
Drainage (underground or covered pucca)	0.011	0.152***	-0.059	-0.035	0.087
	(0.020)	(0.047)	(0.044)	(0.026)	(0.093)
Monthly expenditure per capita (ln)	0.016	-0.042	-0.024	-0.001	-0.075
	(0.031)	(0.040)	(0.021)	(0.037)	(0.051)
Female head	0.011	-0.050	-0.038*	-0.013	-0.059***
	(0.021)	(0.033)	(0.023)	(0.009)	(0.010)
Age head	-0.000	-0.002**	-0.000	-0.000	0.000
	(0.000)	(0.001)	(0.000)	(0.000)	(0.000)
Household size	0.010***	0.001	0.021***	0.013**	0.006
	(0.004)	(0.008)	(0.006)	(0.005)	(0.011)
No. of males	-0.002	0.011	-0.000	-0.005	-0.005
	(0.006)	(0.012)	(0.012)	(0.004)	(0.011)
No. of children	-0.009**	-0.016**	-0.037***	-0.000	-0.002
	(0.004)	(0.008)	(0.010)	(0.010)	(0.012)
No. of elders	0.004	-0.007	-0.021	-0.007	0.005
	(0.005)	(0.012)	(0.016)	(0.009)	(0.007)
Illiterate head	0.001	0.045**	-0.004	-0.025**	-0.034
	(0.013)	(0.019)	(0.017)	(0.012)	(0.036)
High school head	-0.025**	0.003	-0.006	0.031**	-0.016
	(0.011)	(0.023)	(0.019)	(0.014)	(0.016)
Diploma head	0.058*	-0.040	0.007	-0.101**	-0.069
	(0.032)	(0.197)	(0.068)	(0.046)	(0.084)
Uni and above head	-0.035***	0.064	0.001	0.071*	0.013
	(0.013)	(0.040)	(0.030)	(0.043)	(0.033)
Scheduled tribes	-0.037**	-0.172***	-0.006	0.003	0.108*
	(0.016)	(0.034)	(0.019)	(0.025)	(0.063)
Scheduled castes	-0.003	-0.042*	0.005	-0.013	-0.004
	(0.006)	(0.023)	(0.014)	(0.014)	(0.016)
Hindu	0.014	-0.054	-0.019	0.003	0.067
	(0.018)	(0.059)	(0.046)	(0.058)	(0.041)
Islam	0.050	-0.044	-0.006	-0.059	0.069*
	(0.039)	(0.065)	(0.053)	(0.061)	(0.040)
Christianity	-0.026	-0.127	0.012	0.106	-0.075
	(0.019)	(0.129)	(0.113)	(0.077)	(0.093)
Sikhism	0.066	0.058	0.073	-0.058	0.099
	(0.083)	(0.094)	(0.115)	(0.064)	(0.097)
Buddhism	-0.071***	-0.499***	-0.092	-0.142***	-0.337***
	(0.021)	(0.074)	(0.073)	(0.049)	(0.049)
constant	-0.048	1.026***	0.373***	0.124	0.840**
	(0.230)	(0.323)	(0.140)	(0.297)	(0.395)
Number of observations	8615	8615	8615	8615	8615
R^2	0.009	0.021	0.014	0.012	0.009
Adjusted R^2	0.006	0.019	0.012	0.010	0.006
Under-identification	0.136	0.136	0.136	0.136	0.136
Over-identification	N/A	N/A	N/A	N/A	N/A
Weak identification	94.450	94.450	94.450	94.450	94.450

Robust standard errors clustered at state levels; N/A: Not applicable; *** p<0.01, ** p<0.05, * p<0.1.

Supplementary Table S5H: Impacts of adopting only arranged garbage collection on health problems (IV regressions)

	Skin problems	Fly and mosquito problems	Stomach problems	Malaria problems	Other problems
	(1)	(2)	(3)	(4)	(5)
Arranged garbage collection	-0.108	-0.226	-0.136	-0.047	-0.282
	(0.105)	(0.258)	(0.174)	(0.077)	(0.216)
Monthly expenditure per capita (ln)	0.012	-0.019	-0.016	0.003	-0.038
	(0.039)	(0.045)	(0.033)	(0.043)	(0.059)
Female head	0.009	-0.034	-0.033*	-0.011	-0.066***
	(0.019)	(0.032)	(0.020)	(0.008)	(0.013)
Age head	-0.000	-0.001**	-0.000	0.000	0.000
	(0.000)	(0.001)	(0.000)	(0.000)	(0.001)
Household size	0.012**	0.004	0.023***	0.016***	0.007
	(0.005)	(0.007)	(0.007)	(0.006)	(0.010)
No. of males	-0.005	0.013	0.000	-0.006	0.001
	(0.007)	(0.010)	(0.013)	(0.005)	(0.009)
No. of children	-0.011***	-0.024*	-0.036***	-0.003	-0.009
	(0.004)	(0.013)	(0.008)	(0.011)	(0.011)
No. of elders	-0.004	-0.016	-0.023	-0.005	-0.002
	(0.005)	(0.012)	(0.016)	(0.008)	(0.008)
Illiterate head	0.002	0.052***	0.001	-0.022*	-0.038
	(0.011)	(0.017)	(0.016)	(0.012)	(0.036)
High school head	-0.014	-0.001	-0.011	0.027*	-0.018
	(0.014)	(0.025)	(0.022)	(0.016)	(0.017)
Diploma head	0.073***	0.064	0.021	-0.064*	-0.009
	(0.025)	(0.145)	(0.045)	(0.037)	(0.061)
Uni and above head	-0.002	0.079	-0.002	0.085*	0.014
	(0.019)	(0.049)	(0.025)	(0.051)	(0.047)
Scheduled tribes	-0.041**	-0.175***	-0.009	0.008	0.084
	(0.019)	(0.040)	(0.027)	(0.026)	(0.061)
Scheduled castes	-0.001	-0.032	0.011	-0.016	0.002
	(0.005)	(0.020)	(0.015)	(0.012)	(0.016)
Hindu	0.008	-0.067	-0.018	-0.017	0.074
	(0.022)	(0.061)	(0.047)	(0.061)	(0.051)
Islam	0.040	-0.062	-0.014	-0.076	0.068*
	(0.039)	(0.070)	(0.049)	(0.064)	(0.040)
Christianity	-0.035*	-0.128	0.005	0.075	-0.082
	(0.019)	(0.131)	(0.108)	(0.070)	(0.093)
Sikhism	0.053	0.012	0.062	-0.078	0.064
	(0.091)	(0.082)	(0.121)	(0.066)	(0.106)
Buddhism	-0.085***	-0.509***	-0.099	-0.167***	-0.370***
	(0.029)	(0.092)	(0.076)	(0.052)	(0.054)
constant	-0.001	0.886***	0.305	0.100	0.594
	(0.280)	(0.322)	(0.219)	(0.349)	(0.436)
Number of observations	8944	8944	8944	8944	8944
R^2	-0.000	0.014	0.013	0.012	-0.003
Adjusted R^2	-0.002	0.012	0.011	0.010	-0.005
Under-identification	0.401	0.401	0.401	0.401	0.401
Over-identification	N/A	N/A	N/A	N/A	N/A
Weak identification	31.338	31.338	31.338	31.338	31.338

Robust standard errors clustered at state levels; N/A: Not applicable; *** $p<0.01$, ** $p<0.05$, * $p<0.1$.

Supplementary Table S6A: Impacts of adopting drinking water (tap) along with another sanitation practice on health problems (IV regressions)

	Skin problems	Fly and mosquito problems	Stomach problems	Malaria problems	Other problems
	(1)	(2)	(3)	(4)	(5)
Drinking water (tap)	-0.059**	-0.133	-0.057	0.064	-0.167***
	(0.027)	(0.083)	(0.046)	(0.075)	(0.037)
Monthly expenditure per capita (ln)	0.010	-0.049	-0.023	-0.059	-0.033
	(0.011)	(0.049)	(0.029)	(0.041)	(0.046)
Female head	0.004	-0.032	-0.026*	-0.011	-0.046***
	(0.010)	(0.020)	(0.015)	(0.008)	(0.013)
Age head	-0.000	-0.002***	-0.000	-0.001	0.001*
	(0.000)	(0.001)	(0.000)	(0.000)	(0.000)
Household size	0.010***	0.001	0.019***	0.011***	0.010
	(0.003)	(0.005)	(0.005)	(0.003)	(0.007)
No. of males	-0.004	0.012	0.002	0.001	-0.006
	(0.003)	(0.008)	(0.008)	(0.004)	(0.008)
No. of children	-0.007***	-0.012	-0.029***	-0.007	0.006
	(0.003)	(0.008)	(0.009)	(0.011)	(0.008)
No. of elders	-0.004	-0.005	-0.016**	-0.010	0.009
	(0.003)	(0.009)	(0.008)	(0.008)	(0.006)
Illiterate head	-0.002	0.065***	0.004	0.015	-0.036**
	(0.007)	(0.015)	(0.014)	(0.013)	(0.018)
High school head	0.002	-0.034	-0.024**	-0.002	-0.013
	(0.010)	(0.022)	(0.010)	(0.010)	(0.019)
Diploma head	0.001	0.109	-0.043**	-0.003	-0.131***
	(0.019)	(0.067)	(0.022)	(0.059)	(0.047)
Uni and above head	-0.019***	-0.023	-0.017	-0.012	0.009
	(0.005)	(0.033)	(0.016)	(0.017)	(0.021)
Scheduled tribes	-0.013	-0.139***	0.011	0.028**	0.066
	(0.014)	(0.031)	(0.017)	(0.014)	(0.052)
Scheduled castes	0.007*	-0.013	0.013	0.021*	-0.004
	(0.004)	(0.020)	(0.010)	(0.012)	(0.020)
Hindu	0.007	-0.004	-0.029	-0.019	0.055
	(0.014)	(0.060)	(0.034)	(0.038)	(0.035)
Islam	0.039*	-0.052	-0.015	-0.042	0.041
	(0.022)	(0.072)	(0.037)	(0.038)	(0.026)
Christianity	0.005	-0.017	-0.030	-0.008	-0.017
	(0.018)	(0.101)	(0.057)	(0.055)	(0.051)
Sikhism	0.011	-0.134*	0.073*	0.014	0.270***
	(0.024)	(0.073)	(0.041)	(0.036)	(0.041)
Buddhism	-0.008	-0.151*	-0.064	-0.132***	-0.028
	(0.019)	(0.091)	(0.069)	(0.038)	(0.065)
constant	-0.005	1.010**	0.348*	0.521*	0.531
	(0.073)	(0.396)	(0.211)	(0.285)	(0.348)
Number of observations	18240	18240	18240	18240	18240
R^2	0.019	0.056	0.036	-0.004	0.042
Adjusted R^2	0.018	0.055	0.035	-0.005	0.041
Under-identification	0.009	0.009	0.009	0.009	0.009
Over-identification	0.721	0.452	0.351	0.158	0.198
Weak identification	79.390	79.390	79.390	79.390	79.390

Robust standard errors clustered at state levels; *** p<0.01, ** p<0.05, * p<0.1.

Supplementary Table S6B: Impacts of adopting non-drinking water (tap) along with another sanitation practice on health problems (IV regressions)

	Skin problems	Fly and mosquito problems	Stomach problems	Malaria problems	Other problems
	(1)	(2)	(3)	(4)	(5)
Non-drinking water (tap)	-0.057**	-0.099	-0.034	0.084	-0.162***
	(0.029)	(0.118)	(0.065)	(0.095)	(0.057)
Monthly expenditure per capita (ln)	0.011	-0.079	-0.031	-0.067	-0.023
	(0.010)	(0.071)	(0.037)	(0.051)	(0.049)
Female head	0.009	-0.029	-0.030*	-0.019**	-0.036**
	(0.011)	(0.023)	(0.016)	(0.008)	(0.015)
Age head	-0.000	-0.002***	-0.000	-0.001*	0.001
	(0.000)	(0.001)	(0.000)	(0.000)	(0.001)
Household size	0.009***	-0.004	0.017***	0.010***	0.008
	(0.002)	(0.006)	(0.004)	(0.004)	(0.007)
No. of males	-0.003	0.018**	0.002	0.002	-0.000
	(0.003)	(0.008)	(0.008)	(0.005)	(0.008)
No. of children	-0.006*	-0.015*	-0.030***	-0.008	0.004
	(0.003)	(0.008)	(0.010)	(0.012)	(0.008)
No. of elders	-0.005**	-0.006	-0.016**	-0.007	0.013**
	(0.002)	(0.010)	(0.008)	(0.009)	(0.006)
Illiterate head	-0.005	0.063***	0.013	0.016	-0.036*
	(0.008)	(0.023)	(0.017)	(0.017)	(0.021)
High school head	0.002	-0.029*	-0.024**	-0.005	-0.021
	(0.013)	(0.016)	(0.012)	(0.012)	(0.021)
Diploma head	0.005	0.059	-0.053***	-0.051	-0.123***
	(0.024)	(0.076)	(0.018)	(0.041)	(0.047)
Uni and above head	-0.021***	-0.036	-0.025	-0.024	0.023
	(0.007)	(0.039)	(0.021)	(0.019)	(0.027)
Scheduled tribes	-0.015	-0.128***	0.015	0.030**	0.076
	(0.013)	(0.032)	(0.017)	(0.014)	(0.053)
Scheduled castes	0.012**	-0.008	0.019	0.024**	-0.002
	(0.005)	(0.021)	(0.012)	(0.012)	(0.019)
Hindu	0.009	-0.003	-0.037	-0.008	0.060
	(0.016)	(0.063)	(0.034)	(0.029)	(0.037)
Islam	0.044*	-0.036	-0.025	-0.030	0.050*
	(0.025)	(0.076)	(0.036)	(0.031)	(0.028)
Christianity	0.007	-0.086	-0.027	0.046	-0.013
	(0.018)	(0.094)	(0.061)	(0.049)	(0.061)
Sikhism	0.020	-0.147*	0.055	0.023	0.247***
	(0.029)	(0.086)	(0.044)	(0.036)	(0.043)
Buddhism	-0.031	-0.217*	-0.106*	-0.131***	-0.058
	(0.024)	(0.114)	(0.063)	(0.045)	(0.073)
constant	-0.015	1.228**	0.414	0.578	0.456
	(0.078)	(0.560)	(0.273)	(0.358)	(0.365)
Number of observations	17685	17685	17685	17685	17685
R^2	0.016	0.061	0.028	-0.009	0.032
Adjusted R^2	0.015	0.060	0.027	-0.010	0.031
Under-identification	0.012	0.012	0.012	0.012	0.012
Over-identification	0.714	0.633	0.211	0.398	0.089
Weak identification	53.247	53.247	53.247	53.247	53.247

Robust standard errors clustered at state levels; *** $p<0.01$, ** $p<0.05$, * $p<0.1$.

Supplementary Table S6C: Impacts of adopting treated drinking water along with another sanitation practice on health problems (IV regressions)

	Skin problems	Fly and mosquito problems	Stomach problems	Malaria problems	Other problems
	(1)	(2)	(3)	(4)	(5)
Treated drinking water	-0.045*	-0.094	-0.021	0.020	-0.085**
	(0.025)	(0.068)	(0.033)	(0.033)	(0.039)
Monthly expenditure per capita (ln)	0.007	-0.071*	-0.032	-0.043*	-0.049
	(0.011)	(0.041)	(0.026)	(0.026)	(0.053)
Female head	0.001	-0.035	-0.026**	-0.029***	-0.042***
	(0.009)	(0.022)	(0.013)	(0.006)	(0.015)
Age head	-0.000	-0.002***	-0.000	-0.000	0.000
	(0.000)	(0.001)	(0.000)	(0.000)	(0.001)
Household size	0.009***	-0.003	0.019***	0.010***	0.008
	(0.002)	(0.006)	(0.003)	(0.002)	(0.006)
No. of males	-0.000	0.003	0.005	0.002	0.004
	(0.003)	(0.006)	(0.006)	(0.005)	(0.008)
No. of children	-0.008***	-0.006	-0.025***	-0.004	-0.010
	(0.002)	(0.008)	(0.008)	(0.007)	(0.009)
No. of elders	-0.005	0.007	-0.023***	-0.003	0.000
	(0.004)	(0.010)	(0.006)	(0.007)	(0.009)
Illiterate head	-0.001	0.051**	0.009	0.014*	-0.018
	(0.006)	(0.024)	(0.015)	(0.008)	(0.025)
High school head	-0.014**	-0.047**	-0.002	-0.006	-0.013
	(0.006)	(0.020)	(0.014)	(0.012)	(0.021)
Diploma head	-0.010	-0.023	-0.051**	-0.035	-0.029
	(0.014)	(0.045)	(0.021)	(0.025)	(0.043)
Uni and above head	-0.023***	-0.051*	-0.033***	-0.004	0.018
	(0.008)	(0.028)	(0.011)	(0.014)	(0.026)
Scheduled tribes	-0.006	-0.078**	0.024	0.011	0.067
	(0.008)	(0.031)	(0.020)	(0.014)	(0.041)
Scheduled castes	0.005	0.032	0.008	0.011	0.004
	(0.004)	(0.021)	(0.009)	(0.009)	(0.016)
Hindu	-0.018	0.023	-0.030	-0.013	0.006
	(0.015)	(0.050)	(0.025)	(0.036)	(0.031)
Islam	0.018	0.006	-0.013	-0.036	0.028
	(0.019)	(0.062)	(0.036)	(0.037)	(0.044)
Christianity	-0.018	-0.091	0.046	-0.031	-0.053
	(0.020)	(0.071)	(0.056)	(0.032)	(0.062)
Sikhism	-0.012	-0.065	0.042	0.014	0.129**
	(0.025)	(0.065)	(0.037)	(0.030)	(0.051)
Buddhism	-0.041*	-0.018	-0.067	-0.043	-0.109**
	(0.023)	(0.086)	(0.043)	(0.042)	(0.049)
constant	0.037	1.156***	0.407**	0.408**	0.697*
	(0.082)	(0.331)	(0.177)	(0.202)	(0.404)
Number of observations	28227	28227	28227	28227	28227
R^2	0.015	0.050	0.022	0.010	0.022
Adjusted R^2	0.014	0.049	0.022	0.009	0.021
Under-identification	0.009	0.009	0.009	0.009	0.009
Over-identification	0.845	0.449	0.653	0.240	0.249
Weak identification	494.663	494.663	494.663	494.663	494.663

Robust standard errors clustered at state levels; *** p<0.01, ** p<0.05, * p<0.1.

Supplementary Table S6D: Impacts of adopting wash-hand-before-meal-with-soap along with another sanitation practice on health problems (IV regressions)

	Skin problems	Fly and mosquito problems	Stomach problems	Malaria problems	Other problems
	(1)	(2)	(3)	(4)	(5)
Wash hand before meal with soap	-0.045** (0.019)	-0.147** (0.066)	-0.071*** (0.023)	0.019 (0.040)	-0.112*** (0.032)
Monthly expenditure per capita (ln)	0.012 (0.011)	-0.046 (0.040)	-0.001 (0.020)	-0.047** (0.021)	-0.026 (0.041)
Female head	0.014 (0.010)	-0.023 (0.018)	-0.015 (0.012)	-0.023*** (0.007)	-0.051*** (0.013)
Age head	-0.000 (0.000)	-0.002*** (0.001)	-0.000 (0.000)	-0.000 (0.000)	0.001 (0.001)
Household size	0.011*** (0.002)	0.007 (0.005)	0.018*** (0.003)	0.011*** (0.003)	0.010* (0.006)
No. of males	0.000 (0.003)	0.002 (0.008)	0.004 (0.007)	0.000 (0.004)	0.001 (0.008)
No. of children	-0.005 (0.003)	-0.004 (0.006)	-0.020*** (0.006)	-0.008 (0.007)	-0.008 (0.009)
No. of elders	-0.004 (0.007)	-0.000 (0.008)	-0.016* (0.009)	-0.012** (0.005)	-0.002 (0.008)
Illiterate head	-0.003 (0.006)	0.038* (0.020)	-0.005 (0.012)	0.006 (0.008)	-0.029 (0.024)
High school head	-0.006 (0.008)	-0.014 (0.016)	-0.017 (0.015)	-0.013** (0.006)	-0.020 (0.020)
Diploma head	0.018 (0.017)	-0.034 (0.050)	-0.036** (0.017)	-0.054*** (0.016)	-0.063* (0.037)
Uni and above head	-0.021*** (0.007)	-0.024 (0.023)	-0.025** (0.012)	-0.017 (0.013)	-0.009 (0.021)
Scheduled tribes	-0.015 (0.013)	-0.130*** (0.027)	0.018 (0.018)	-0.001 (0.014)	0.074 (0.047)
Scheduled castes	0.005 (0.008)	-0.012 (0.018)	0.012 (0.011)	0.007 (0.009)	0.016 (0.016)
Hindu	0.003 (0.017)	-0.011 (0.051)	-0.031 (0.029)	-0.004 (0.037)	0.020 (0.029)
Islam	0.026 (0.019)	-0.066 (0.055)	-0.032 (0.028)	-0.038 (0.042)	0.065** (0.030)
Christianity	-0.013 (0.018)	-0.099 (0.075)	-0.020 (0.046)	-0.028 (0.034)	-0.098* (0.055)
Sikhism	0.020 (0.018)	-0.146** (0.061)	0.074** (0.034)	0.041* (0.025)	0.102*** (0.037)
Buddhism	-0.007 (0.018)	-0.065 (0.095)	-0.079* (0.041)	-0.022 (0.036)	-0.125* (0.072)
constant	-0.023 (0.086)	0.987*** (0.320)	0.188 (0.153)	0.436*** (0.148)	0.485 (0.312)
Number of observations	24791	24791	24791	24791	24791
R^2	0.013	0.048	0.021	0.010	0.028
Adjusted R^2	0.012	0.048	0.021	0.010	0.028
Under-identification	0.004	0.004	0.004	0.004	0.004
Over-identification	0.545	0.364	0.218	0.390	0.541
Weak identification	433.568	433.568	433.568	433.568	433.568

Robust standard errors clustered at state levels; *** $p<0.01$, ** $p<0.05$, * $p<0.1$.

Supplementary Table S6E: Impacts of adopting a toilet (exclusive use) along with another sanitation practice on health problems (IV regressions)

	Skin problems	Fly and mosquito problems	Stomach problems	Malaria problems	Other problems
	(1)	(2)	(3)	(4)	(5)
Toilet (exclusive use)	-0.028	-0.114***	-0.053***	0.014	-0.061*
	(0.019)	(0.041)	(0.018)	(0.024)	(0.034)
Monthly expenditure per capita (ln)	-0.004	-0.072**	-0.024	-0.023	-0.056
	(0.010)	(0.033)	(0.018)	(0.018)	(0.041)
Female head	0.003	-0.032**	-0.021**	-0.020***	-0.031**
	(0.005)	(0.014)	(0.009)	(0.005)	(0.013)
Age head	-0.000	-0.001***	-0.000	-0.000	0.000
	(0.000)	(0.000)	(0.000)	(0.000)	(0.001)
Household size	0.009***	0.002	0.017***	0.014***	0.009**
	(0.002)	(0.004)	(0.003)	(0.003)	(0.005)
No. of males	0.004	0.013*	0.011**	0.001	0.017***
	(0.003)	(0.007)	(0.005)	(0.004)	(0.005)
No. of children	-0.007***	-0.013*	-0.020***	-0.005	-0.014**
	(0.002)	(0.008)	(0.004)	(0.004)	(0.007)
No. of elders	-0.003	-0.008	-0.020***	-0.008**	-0.012
	(0.003)	(0.008)	(0.006)	(0.004)	(0.008)
Illiterate head	0.006	0.035**	0.002	0.018***	-0.018
	(0.006)	(0.015)	(0.012)	(0.005)	(0.025)
High school head	-0.005	-0.015	-0.003	-0.006	-0.001
	(0.008)	(0.018)	(0.010)	(0.007)	(0.019)
Diploma head	0.005	0.038	-0.041***	-0.010	-0.078**
	(0.014)	(0.046)	(0.013)	(0.025)	(0.034)
Uni and above head	-0.012*	0.003	-0.016*	-0.016	0.023
	(0.007)	(0.030)	(0.008)	(0.013)	(0.017)
Scheduled tribes	-0.016	-0.112***	0.019	0.020	0.044
	(0.011)	(0.031)	(0.019)	(0.015)	(0.040)
Scheduled castes	0.002	0.005	0.002	0.012	0.014
	(0.004)	(0.016)	(0.008)	(0.010)	(0.014)
Hindu	-0.010	0.017	-0.038*	-0.019	0.016
	(0.010)	(0.038)	(0.021)	(0.030)	(0.029)
Islam	0.022*	-0.009	-0.014	-0.033	0.076**
	(0.012)	(0.048)	(0.026)	(0.034)	(0.033)
Christianity	-0.024**	-0.073	0.016	-0.026	-0.056
	(0.012)	(0.066)	(0.044)	(0.028)	(0.055)
Sikhism	0.005	-0.133**	0.072***	0.009	0.148***
	(0.015)	(0.059)	(0.027)	(0.022)	(0.041)
Buddhism	-0.036**	-0.054	-0.050	-0.056*	-0.138***
	(0.016)	(0.074)	(0.032)	(0.030)	(0.048)
constant	0.099	1.142***	0.349***	0.246*	0.699**
	(0.087)	(0.269)	(0.130)	(0.144)	(0.307)
Number of observations	47984	47984	47984	47984	47984
R^2	0.012	0.033	0.023	0.012	0.022
Adjusted R^2	0.012	0.033	0.023	0.012	0.021
Under-identification	0.012	0.012	0.012	0.012	0.012
Over-identification	0.628	0.981	0.495	0.569	0.328
Weak identification	1740.919	1740.919	1740.919	1740.919	1740.919

Robust standard errors clustered at state levels; *** $p<0.01$, ** $p<0.05$, * $p<0.1$.

Supplementary Table S6F: Impacts of adopting bathroom (exclusive use) along with another sanitation practice on health problems (IV regressions)

	Skin problems	Fly and mosquito problems	Stomach problems	Malaria problems	Other problems
	(1)	(2)	(3)	(4)	(5)
Bathroom (exclusive use)	-0.032	-0.128***	-0.056***	0.011	-0.094***
	(0.020)	(0.039)	(0.020)	(0.026)	(0.031)
Monthly expenditure per capita (ln)	-0.004	-0.071**	-0.030*	-0.024	-0.047
	(0.012)	(0.030)	(0.018)	(0.019)	(0.040)
Female head	0.002	-0.028**	-0.010	-0.010**	-0.023*
	(0.005)	(0.014)	(0.008)	(0.005)	(0.014)
Age head	-0.000	-0.001***	-0.000	-0.001**	0.000
	(0.000)	(0.000)	(0.000)	(0.000)	(0.001)
Household size	0.009***	0.002	0.017***	0.013***	0.009*
	(0.002)	(0.005)	(0.003)	(0.003)	(0.005)
No. of males	0.003	0.012	0.010	0.001	0.017***
	(0.003)	(0.007)	(0.007)	(0.003)	(0.005)
No. of children	-0.008***	-0.012	-0.021***	-0.005	-0.011*
	(0.002)	(0.008)	(0.005)	(0.004)	(0.006)
No. of elders	-0.003	-0.006	-0.021***	-0.007*	-0.007
	(0.004)	(0.007)	(0.006)	(0.004)	(0.006)
Illiterate head	0.006	0.037**	-0.002	0.014**	-0.029
	(0.007)	(0.018)	(0.011)	(0.006)	(0.027)
High school head	-0.006	-0.005	0.000	-0.002	-0.001
	(0.009)	(0.016)	(0.011)	(0.006)	(0.017)
Diploma head	0.018	0.028	-0.049***	-0.032*	-0.075**
	(0.018)	(0.048)	(0.013)	(0.017)	(0.034)
Uni and above head	-0.014**	0.003	-0.011	-0.016	0.021
	(0.007)	(0.026)	(0.010)	(0.014)	(0.018)
Scheduled tribes	-0.016	-0.106***	0.017	0.019	0.045
	(0.011)	(0.032)	(0.020)	(0.016)	(0.043)
Scheduled castes	0.002	-0.000	0.003	0.010	0.015
	(0.006)	(0.019)	(0.010)	(0.010)	(0.014)
Hindu	-0.015	0.015	-0.023	-0.008	0.032
	(0.013)	(0.046)	(0.024)	(0.025)	(0.027)
Islam	0.017	-0.016	0.003	-0.019	0.076**
	(0.015)	(0.053)	(0.029)	(0.031)	(0.038)
Christianity	-0.029*	-0.073	0.033	-0.015	-0.044
	(0.016)	(0.075)	(0.052)	(0.028)	(0.053)
Sikhism	0.006	-0.122*	0.086***	0.016	0.169***
	(0.018)	(0.065)	(0.030)	(0.019)	(0.039)
Buddhism	-0.038*	-0.058	-0.041	-0.031	-0.100**
	(0.020)	(0.080)	(0.035)	(0.028)	(0.045)
constant	0.109	1.137***	0.385***	0.264*	0.633**
	(0.098)	(0.264)	(0.135)	(0.149)	(0.308)
Number of observations	41133	41133	41133	41133	41133
R^2	0.014	0.037	0.026	0.011	0.024
Adjusted R^2	0.013	0.037	0.025	0.011	0.024
Under-identification	0.005	0.005	0.005	0.005	0.005
Over-identification	0.427	0.973	0.474	0.393	0.389
Weak identification	1320.573	1320.573	1320.573	1320.573	1320.573

Robust standard errors clustered at state levels; *** p<0.01, ** p<0.05, * p<0.1.

Supplementary Table S6G: Impacts of adopting drainage (underground or covered pucca) along with another sanitation practice on health problems (IV regressions)

	Skin problems	Fly and mosquito problems	Stomach problems	Malaria problems	Other problems
	(1)	(2)	(3)	(4)	(5)
Drainage (underground or covered pucca)	-0.020	-0.083	-0.042	0.052	-0.096**
	(0.038)	(0.098)	(0.043)	(0.053)	(0.047)
Monthly expenditure per capita (ln)	-0.005	-0.121**	-0.038	-0.044	-0.043
	(0.009)	(0.056)	(0.024)	(0.028)	(0.045)
Female head	0.013	-0.054***	-0.031**	-0.026***	-0.048**
	(0.008)	(0.020)	(0.012)	(0.007)	(0.021)
Age head	-0.000*	-0.001**	-0.000	-0.000	0.001
	(0.000)	(0.000)	(0.000)	(0.000)	(0.001)
Household size	0.011***	0.000	0.020***	0.011***	0.008
	(0.002)	(0.005)	(0.004)	(0.003)	(0.005)
No. of males	-0.001	0.012*	0.006	-0.001	0.008
	(0.003)	(0.007)	(0.005)	(0.004)	(0.005)
No. of children	-0.007**	-0.016**	-0.029***	-0.002	-0.014**
	(0.003)	(0.008)	(0.007)	(0.008)	(0.006)
No. of elders	-0.002	-0.015**	-0.016**	-0.013*	-0.002
	(0.004)	(0.007)	(0.007)	(0.007)	(0.010)
Illiterate head	0.009	0.054***	0.009	0.013	-0.017
	(0.007)	(0.016)	(0.014)	(0.008)	(0.030)
High school head	-0.027**	-0.008	-0.003	-0.007	-0.000
	(0.013)	(0.013)	(0.017)	(0.008)	(0.024)
Diploma head	-0.006	0.033	-0.067***	-0.091***	-0.042
	(0.015)	(0.071)	(0.019)	(0.015)	(0.053)
Uni and above head	-0.011	0.032	-0.005	-0.026	0.019
	(0.008)	(0.026)	(0.010)	(0.017)	(0.024)
Scheduled tribes	-0.028*	-0.144***	-0.006	0.027	0.076
	(0.015)	(0.032)	(0.020)	(0.017)	(0.052)
Scheduled castes	0.009	-0.009	0.010	0.020*	0.001
	(0.006)	(0.019)	(0.010)	(0.012)	(0.017)
Hindu	0.017	-0.031	-0.012	-0.023	0.079**
	(0.010)	(0.054)	(0.024)	(0.047)	(0.031)
Islam	0.041**	-0.056	0.007	-0.044	0.105***
	(0.018)	(0.054)	(0.028)	(0.049)	(0.037)
Christianity	-0.021	-0.091	-0.033	-0.050	-0.072
	(0.017)	(0.090)	(0.049)	(0.040)	(0.052)
Sikhism	0.045	-0.107	0.138***	0.019	0.192***
	(0.030)	(0.078)	(0.029)	(0.029)	(0.048)
Buddhism	0.001	-0.189**	-0.108***	-0.085**	-0.166***
	(0.025)	(0.093)	(0.033)	(0.037)	(0.041)
constant	0.088	1.535***	0.440***	0.415**	0.572*
	(0.063)	(0.423)	(0.166)	(0.185)	(0.345)
Number of observations	19786	19786	19786	19786	19786
R^2	0.015	0.049	0.032	0.003	0.029
Adjusted R^2	0.014	0.048	0.031	0.002	0.028
Under-identification	0.010	0.010	0.010	0.010	0.010
Over-identification	0.220	0.581	0.306	0.486	0.545
Weak identification	198.680	198.680	198.680	198.680	198.680

Robust standard errors clustered at state levels; *** p<0.01, ** p<0.05, * p<0.1.

Supplementary Table S6H: Impacts of adopting arranged garbage collection along with another sanitation practice on health problems (IV regressions)

	Skin problems	Fly and mosquito problems	Stomach problems	Malaria problems	Other problems
	(1)	(2)	(3)	(4)	(5)
Arranged garbage collection	-0.052*	-0.106	-0.055*	0.039	-0.048
	(0.030)	(0.066)	(0.033)	(0.057)	(0.054)
Monthly expenditure per capita (ln)	-0.001	-0.081	-0.030	-0.059**	-0.071
	(0.012)	(0.049)	(0.027)	(0.030)	(0.060)
Female head	0.005	-0.052***	-0.025*	-0.022***	-0.035*
	(0.008)	(0.018)	(0.015)	(0.005)	(0.018)
Age head	-0.000	-0.002***	0.000	-0.000	0.000
	(0.000)	(0.001)	(0.000)	(0.000)	(0.000)
Household size	0.008***	0.006	0.018***	0.012***	0.012*
	(0.003)	(0.006)	(0.004)	(0.003)	(0.007)
No. of males	0.001	0.006	0.008	0.000	0.012
	(0.004)	(0.010)	(0.009)	(0.005)	(0.009)
No. of children	-0.006***	-0.011	-0.023***	-0.004	-0.010
	(0.002)	(0.007)	(0.006)	(0.008)	(0.010)
No. of elders	-0.003	-0.017**	-0.020**	-0.011	0.001
	(0.004)	(0.007)	(0.009)	(0.008)	(0.008)
Illiterate head	-0.001	0.056***	0.004	0.014	-0.015
	(0.008)	(0.019)	(0.013)	(0.010)	(0.022)
High school head	-0.014**	-0.031	0.021	0.014	0.003
	(0.006)	(0.024)	(0.014)	(0.011)	(0.030)
Diploma head	0.024	0.092	-0.019	-0.003	-0.051
	(0.028)	(0.074)	(0.025)	(0.054)	(0.035)
Uni and above head	-0.008	0.051*	-0.026**	-0.008	0.057*
	(0.010)	(0.029)	(0.012)	(0.019)	(0.030)
Scheduled tribes	-0.018	-0.129***	0.003	0.027*	0.072
	(0.013)	(0.039)	(0.019)	(0.015)	(0.058)
Scheduled castes	0.000	-0.002	0.004	-0.001	-0.009
	(0.005)	(0.020)	(0.011)	(0.010)	(0.013)
Hindu	0.006	-0.003	-0.043	-0.051	0.039
	(0.015)	(0.068)	(0.036)	(0.045)	(0.033)
Islam	0.040*	-0.005	-0.030	-0.054	0.088**
	(0.022)	(0.070)	(0.046)	(0.052)	(0.040)
Christianity	0.004	-0.091	0.010	-0.064	-0.057
	(0.021)	(0.108)	(0.063)	(0.044)	(0.055)
Sikhism	0.032	-0.063	0.070*	0.054*	0.315***
	(0.023)	(0.092)	(0.042)	(0.028)	(0.059)
Buddhism	0.009	-0.078	-0.022	-0.067	-0.069
	(0.027)	(0.115)	(0.061)	(0.058)	(0.083)
constant	0.079	1.233***	0.372**	0.540***	0.783*
	(0.086)	(0.411)	(0.185)	(0.199)	(0.456)
Number of observations	19913	19913	19913	19913	19913
R^2	0.017	0.048	0.029	0.006	0.028
Adjusted R^2	0.017	0.047	0.028	0.005	0.027
Under-identification	0.006	0.006	0.006	0.006	0.006
Over-identification	0.521	0.464	0.095	0.424	0.251
Weak identification	128.844	128.844	128.844	128.844	128.844

Robust standard errors clustered at state levels; *** p<0.01, ** p<0.05, * p<0.1.

Supplementary Table S7A: Impacts of adopting a different number of sanitation practices on health problems: Adopting one sanitation practice (IV regressions)

	Skin problems	Fly and mosquito problems	Stomach problems	Malaria problems	Other problems
	(1)	(2)	(3)	(4)	(5)
Adopting one sanitation practice	-0.074	-0.119	-0.129	0.175**	0.262
	(0.123)	(0.196)	(0.125)	(0.075)	(0.236)
Monthly expenditure per capita (ln)	0.023	-0.020	-0.012	-0.048	-0.134
	(0.021)	(0.068)	(0.044)	(0.050)	(0.104)
Female head	0.008	-0.035	-0.023	0.007	-0.041***
	(0.016)	(0.026)	(0.015)	(0.008)	(0.015)
Age head	0.000	-0.001	-0.000	-0.001***	-0.001
	(0.000)	(0.000)	(0.000)	(0.000)	(0.001)
Household size	0.011***	0.004	0.015***	0.012***	0.005
	(0.002)	(0.006)	(0.003)	(0.005)	(0.010)
No. of males	0.001	0.010	0.010**	0.003	0.007
	(0.003)	(0.007)	(0.005)	(0.005)	(0.009)
No. of children	-0.008*	-0.013**	-0.021***	-0.004	-0.017
	(0.004)	(0.006)	(0.005)	(0.007)	(0.011)
No. of elders	-0.004	-0.008	-0.015	0.001	-0.003
	(0.004)	(0.006)	(0.013)	(0.004)	(0.008)
Illiterate head	-0.014	0.031	-0.015	0.007	0.011
	(0.013)	(0.020)	(0.014)	(0.009)	(0.020)
High school head	-0.013**	0.049**	-0.008	-0.011	-0.020
	(0.006)	(0.025)	(0.014)	(0.012)	(0.020)
Diploma head	0.018	0.008	-0.008	0.036	0.012
	(0.027)	(0.100)	(0.046)	(0.042)	(0.058)
Uni and above head	-0.023	0.047	-0.001	0.016	0.014
	(0.018)	(0.036)	(0.031)	(0.040)	(0.027)
Scheduled tribes	-0.023	-0.116***	0.001	0.031	0.048
	(0.017)	(0.038)	(0.013)	(0.026)	(0.052)
Scheduled castes	-0.001	-0.020	0.003	-0.007	0.004
	(0.006)	(0.014)	(0.010)	(0.016)	(0.017)
Hindu	-0.009	-0.077***	-0.049	-0.058	-0.006
	(0.029)	(0.025)	(0.044)	(0.073)	(0.044)
Islam	0.015	-0.051	-0.044	-0.139*	0.013
	(0.043)	(0.053)	(0.057)	(0.080)	(0.049)
Christianity	-0.052*	-0.108	-0.019	-0.000	-0.077
	(0.030)	(0.082)	(0.067)	(0.072)	(0.102)
Sikhism	0.028	-0.139	0.087	-0.076	0.021
	(0.050)	(0.097)	(0.066)	(0.069)	(0.057)
Buddhism	-0.026	-0.194**	0.042	-0.109	-0.360***
	(0.049)	(0.089)	(0.070)	(0.084)	(0.072)
constant	-0.056	0.873**	0.355	0.414	1.206*
	(0.134)	(0.444)	(0.285)	(0.377)	(0.657)
Number of observations	20438	20438	20438	20438	20438
R^2	-0.005	0.007	-0.005	-0.059	-0.070
Adjusted R^2	-0.006	0.006	-0.006	-0.060	-0.071
Under-identification	0.394	0.394	0.394	0.394	0.394
Over-identification	0.193	0.431	0.418	0.324	0.171
Weak identification	13.661	13.661	13.661	13.661	13.661

Robust standard errors clustered at state levels; *** $p<0.01$, ** $p<0.05$, * $p<0.1$.

Supplementary Table S7B: Impacts of adopting a different number of sanitation practices on health problems: adopting two sanitation practices (IV regressions)

	Skin problems	Fly and mosquito problems	Stomach problems	Malaria problems	Other problems
	(1)	(2)	(3)	(4)	(5)
Adopting two sanitation practices	0.001	-0.107	-0.069	0.125	-0.080
	(0.073)	(0.098)	(0.068)	(0.088)	(0.091)
Monthly expenditure per capita (ln)	-0.000	-0.021	-0.020	-0.047	-0.040
	(0.016)	(0.052)	(0.029)	(0.037)	(0.071)
Female head	0.005	-0.010	-0.026***	-0.017***	-0.037***
	(0.010)	(0.020)	(0.010)	(0.007)	(0.013)
Age head	-0.001*	-0.002**	-0.000	-0.000	0.001*
	(0.000)	(0.001)	(0.000)	(0.000)	(0.000)
Household size	0.012***	0.002	0.020***	0.017***	0.009
	(0.003)	(0.006)	(0.003)	(0.005)	(0.008)
No. of males	-0.002	0.015**	0.004	-0.003	0.015**
	(0.004)	(0.006)	(0.006)	(0.004)	(0.006)
No. of children	-0.007***	-0.008	-0.021***	-0.009	-0.009
	(0.003)	(0.006)	(0.005)	(0.008)	(0.009)
No. of elders	0.003	-0.005	-0.017**	-0.005	-0.018**
	(0.004)	(0.009)	(0.008)	(0.007)	(0.007)
Illiterate head	0.015	0.029*	-0.004	0.018	-0.037
	(0.011)	(0.016)	(0.016)	(0.014)	(0.029)
High school head	-0.016	0.005	0.003	-0.022*	0.027
	(0.016)	(0.024)	(0.014)	(0.013)	(0.024)
Diploma head	0.015	-0.076	0.029	0.011	-0.075*
	(0.028)	(0.098)	(0.036)	(0.039)	(0.040)
Uni and above head	0.003	0.071	0.000	-0.029	0.050
	(0.008)	(0.044)	(0.021)	(0.027)	(0.035)
Scheduled tribes	-0.041***	-0.135***	-0.005	0.027	0.045
	(0.014)	(0.035)	(0.019)	(0.024)	(0.047)
Scheduled castes	-0.008	-0.011	-0.005	0.004	-0.001
	(0.006)	(0.021)	(0.011)	(0.017)	(0.016)
Hindu	-0.000	-0.058	-0.057*	-0.034	0.020
	(0.013)	(0.037)	(0.030)	(0.061)	(0.043)
Islam	0.033	-0.046	-0.030	-0.076	0.092**
	(0.023)	(0.052)	(0.047)	(0.060)	(0.042)
Christianity	-0.021	-0.096	0.014	0.033	-0.041
	(0.026)	(0.091)	(0.068)	(0.053)	(0.080)
Sikhism	0.000	-0.283***	0.063	-0.099*	0.174***
	(0.040)	(0.085)	(0.040)	(0.051)	(0.054)
Buddhism	-0.042	-0.046	-0.034	-0.089	-0.136**
	(0.037)	(0.085)	(0.039)	(0.068)	(0.066)
constant	0.073	0.877**	0.366**	0.400	0.593
	(0.109)	(0.376)	(0.185)	(0.281)	(0.533)
Number of observations	20608	20608	20608	20608	20608
R^2	0.011	0.022	0.016	-0.026	0.016
Adjusted R^2	0.010	0.021	0.015	-0.026	0.015
Under-identification	0.060	0.060	0.060	0.060	0.060
Over-identification	0.707	0.733	0.231	0.352	0.349
Weak identification	44.607	44.607	44.607	44.607	44.607

Robust standard errors clustered at state levels; *** $p<0.01$, ** $p<0.05$, * $p<0.1$.

Supplementary Table S7C: Impacts of adopting a different number of sanitation practices in the households on health problems: adopting three sanitation practices (IV regressions)

	Skin problems	Fly and mosquito problems	Stomach problems	Malaria problems	Other problems
	(1)	(2)	(3)	(4)	(5)
Adopting three sanitation practices	-0.076**	-0.221*	-0.097	0.107	-0.174**
	(0.038)	(0.127)	(0.064)	(0.123)	(0.082)
Monthly expenditure per capita (ln)	0.025	0.004	-0.001	-0.069	0.000
	(0.020)	(0.063)	(0.035)	(0.061)	(0.070)
Female head	0.015*	-0.033*	-0.021*	-0.028***	-0.022*
	(0.009)	(0.018)	(0.011)	(0.008)	(0.012)
Age head	-0.000	-0.001	0.000	-0.001	0.001
	(0.000)	(0.001)	(0.000)	(0.001)	(0.001)
Household size	0.011***	0.006	0.018***	0.008**	0.010
	(0.002)	(0.007)	(0.005)	(0.004)	(0.007)
No. of males	0.003	0.010	0.009	0.004	0.012*
	(0.003)	(0.008)	(0.008)	(0.005)	(0.006)
No. of children	-0.003	-0.009	-0.021***	-0.010	-0.008
	(0.004)	(0.008)	(0.005)	(0.009)	(0.012)
No. of elders	0.002	-0.006	-0.024**	-0.008	-0.004
	(0.006)	(0.012)	(0.011)	(0.006)	(0.009)
Illiterate head	-0.011**	0.014	-0.012	0.018	-0.057**
	(0.005)	(0.024)	(0.012)	(0.018)	(0.027)
High school head	0.008	-0.009	0.012	-0.019	0.019
	(0.013)	(0.034)	(0.015)	(0.015)	(0.033)
Diploma head	0.007	0.138	0.011	-0.012	-0.075*
	(0.019)	(0.102)	(0.021)	(0.077)	(0.045)
Uni and above head	-0.004	0.058	0.026	-0.043	0.079
	(0.008)	(0.065)	(0.021)	(0.034)	(0.048)
Scheduled tribes	-0.023**	-0.145***	-0.001	0.013	0.050
	(0.012)	(0.028)	(0.018)	(0.017)	(0.050)
Scheduled castes	-0.007	-0.024	-0.008	0.013	-0.006
	(0.007)	(0.022)	(0.012)	(0.019)	(0.021)
Hindu	-0.000	-0.007	-0.018	-0.016	0.025
	(0.014)	(0.042)	(0.039)	(0.030)	(0.032)
Islam	0.039**	-0.011	0.020	-0.048	0.102***
	(0.019)	(0.050)	(0.050)	(0.030)	(0.036)
Christianity	-0.016	-0.071	0.068	-0.037	0.002
	(0.017)	(0.082)	(0.070)	(0.030)	(0.075)
Sikhism	0.033	-0.093	0.078	-0.044	0.161**
	(0.028)	(0.095)	(0.051)	(0.046)	(0.070)
Buddhism	-0.028	0.069	-0.003	-0.060	-0.023
	(0.021)	(0.073)	(0.047)	(0.067)	(0.071)
constant	-0.106	0.638	0.177	0.586	0.334
	(0.141)	(0.444)	(0.237)	(0.424)	(0.519)
Number of observations	19387	19387	19387	19387	19387
R^2	0.011	0.028	0.019	-0.027	0.017
Adjusted R^2	0.010	0.027	0.019	-0.028	0.016
Under-identification	0.023	0.023	0.023	0.023	0.023
Over-identification	0.126	0.700	0.546	0.307	0.565
Weak identification	60.265	60.265	60.265	60.265	60.265

Robust standard errors clustered at state levels; *** $p<0.01$, ** $p<0.05$, * $p<0.1$.

Supplementary Table S7D: Impacts of adopting a different number of sanitation practices on health problems: adopting four sanitation practices (IV regressions)

	Skin problems	Fly and mosquito problems	Stomach problems	Malaria problems	Other problems
	(1)	(2)	(3)	(4)	(5)
Adopting four sanitation practices	-0.042	-0.119	0.006	0.045	-0.130*
	(0.037)	(0.120)	(0.069)	(0.109)	(0.070)
Monthly expenditure per capita (ln)	0.009	-0.048	-0.057	-0.064	-0.040
	(0.021)	(0.078)	(0.042)	(0.055)	(0.055)
Female head	0.008	-0.042	-0.026	-0.022***	-0.057***
	(0.011)	(0.030)	(0.018)	(0.006)	(0.012)
Age head	-0.000	-0.001**	-0.001*	-0.001	0.001
	(0.000)	(0.001)	(0.000)	(0.001)	(0.001)
Household size	0.009**	0.003	0.020***	0.010***	0.009
	(0.004)	(0.007)	(0.005)	(0.003)	(0.007)
No. of males	0.003	0.010	0.004	0.002	0.002
	(0.006)	(0.009)	(0.008)	(0.004)	(0.009)
No. of children	-0.009***	-0.008	-0.033***	-0.010	-0.001
	(0.003)	(0.008)	(0.009)	(0.011)	(0.008)
No. of elders	-0.002	0.003	-0.021**	-0.004	0.001
	(0.003)	(0.010)	(0.009)	(0.006)	(0.007)
Illiterate head	0.005	0.047*	0.006	0.004	-0.025
	(0.008)	(0.025)	(0.018)	(0.017)	(0.027)
High school head	-0.010	-0.007	-0.025*	0.004	-0.013
	(0.009)	(0.014)	(0.015)	(0.010)	(0.025)
Diploma head	0.045	-0.009	-0.060**	-0.047*	0.013
	(0.029)	(0.072)	(0.027)	(0.026)	(0.063)
Uni and above head	-0.015*	-0.014	-0.029	-0.008	0.019
	(0.009)	(0.033)	(0.017)	(0.019)	(0.027)
Scheduled tribes	-0.024	-0.130***	0.022	0.010	0.088*
	(0.015)	(0.026)	(0.021)	(0.015)	(0.047)
Scheduled castes	0.003	0.005	0.010	0.007	-0.003
	(0.006)	(0.017)	(0.011)	(0.010)	(0.018)
Hindu	-0.005	-0.035	-0.012	0.011	0.059*
	(0.021)	(0.058)	(0.034)	(0.032)	(0.034)
Islam	0.024	-0.067	0.002	-0.020	0.059
	(0.030)	(0.063)	(0.035)	(0.033)	(0.039)
Christianity	-0.030	-0.136	0.040	-0.014	-0.040
	(0.025)	(0.102)	(0.067)	(0.032)	(0.064)
Sikhism	0.018	-0.249***	0.100**	0.002	0.220***
	(0.032)	(0.085)	(0.042)	(0.035)	(0.066)
Buddhism	-0.029	-0.196*	-0.101	-0.038	-0.153***
	(0.029)	(0.115)	(0.062)	(0.053)	(0.056)
constant	0.009	1.008	0.576*	0.551	0.574
	(0.148)	(0.613)	(0.306)	(0.389)	(0.422)
Number of observations	16528	16528	16528	16528	16528
R^2	0.014	0.053	0.023	0.001	0.033
Adjusted R^2	0.012	0.052	0.022	0.000	0.031
Under-identification	0.011	0.011	0.011	0.011	0.011
Over-identification	0.534	0.571	0.479	0.395	0.161
Weak identification	34.078	34.078	34.078	34.078	34.078

Robust standard errors clustered at state levels; *** $p<0.01$, ** $p<0.05$, * $p<0.1$.

Supplementary Table S7E: Impacts of adopting a different number of sanitation practices on health problems: adopting five sanitation practices (IV regressions)

	Skin problems	Fly and mosquito problems	Stomach problems	Malaria problems	Other problems
	(1)	(2)	(3)	(4)	(5)
Adopting five sanitation practices	-0.027	-0.103*	-0.024	-0.139	-0.124
	(0.050)	(0.056)	(0.080)	(0.088)	(0.168)
Monthly expenditure per capita (ln)	-0.005	-0.094***	-0.034	0.038	-0.029
	(0.037)	(0.032)	(0.036)	(0.055)	(0.074)
Female head	0.001	-0.016	-0.024	-0.008	-0.042*
	(0.014)	(0.025)	(0.019)	(0.010)	(0.024)
Age head	-0.000	-0.003***	-0.000	0.000	0.001
	(0.000)	(0.001)	(0.000)	(0.000)	(0.001)
Household size	0.010**	-0.003	0.019***	0.016***	0.009
	(0.004)	(0.005)	(0.006)	(0.004)	(0.008)
No. of males	-0.002	0.016*	0.004	-0.004	0.002
	(0.005)	(0.009)	(0.011)	(0.006)	(0.008)
No. of children	-0.009***	-0.008	-0.031***	0.004	-0.002
	(0.003)	(0.009)	(0.011)	(0.013)	(0.014)
No. of elders	-0.007*	-0.002	-0.018*	-0.004	0.003
	(0.004)	(0.010)	(0.011)	(0.008)	(0.009)
Illiterate head	0.001	0.065***	0.006	-0.019	-0.035
	(0.014)	(0.017)	(0.019)	(0.019)	(0.044)
High school head	-0.021*	-0.005	-0.029*	0.007	-0.029
	(0.011)	(0.021)	(0.016)	(0.010)	(0.022)
Diploma head	0.041	0.040	-0.073***	-0.027	-0.080
	(0.030)	(0.068)	(0.028)	(0.042)	(0.056)
Uni and above head	-0.013	-0.037	-0.060**	0.019	-0.006
	(0.014)	(0.032)	(0.024)	(0.017)	(0.032)
Scheduled tribes	-0.018	-0.151***	0.013	-0.000	0.090
	(0.018)	(0.031)	(0.019)	(0.022)	(0.055)
Scheduled castes	0.010*	-0.031	0.026	-0.007	-0.006
	(0.005)	(0.020)	(0.016)	(0.013)	(0.017)
Hindu	0.006	0.004	-0.023	-0.005	0.068**
	(0.017)	(0.070)	(0.036)	(0.034)	(0.034)
Islam	0.029	-0.044	-0.021	-0.042	0.058**
	(0.028)	(0.075)	(0.038)	(0.044)	(0.029)
Christianity	-0.018	-0.090	-0.021	0.032	-0.069
	(0.019)	(0.097)	(0.067)	(0.046)	(0.058)
Sikhism	0.028	-0.153**	0.104*	0.046*	0.127**
	(0.024)	(0.077)	(0.056)	(0.026)	(0.062)
Buddhism	0.021	-0.181*	-0.064	-0.046	-0.098
	(0.027)	(0.098)	(0.085)	(0.034)	(0.105)
constant	0.099	1.371***	0.420	-0.157	0.479
	(0.274)	(0.280)	(0.281)	(0.391)	(0.535)
Number of observations	13753	13753	13753	13753	13753
R^2	0.013	0.063	0.025	0.009	0.029
Adjusted R^2	0.012	0.062	0.024	0.008	0.028
Under-identification	0.014	0.014	0.014	0.014	0.014
Over-identification	0.718	0.951	0.121	0.185	0.109
Weak identification	36.446	36.446	36.446	36.446	36.446

Robust standard errors clustered at state levels; *** p<0.01, ** p<0.05, * p<0.1.

Supplementary Table S7F: Impacts of adopting a different number of sanitation practices on health problems: adopting six sanitation practices (IV regressions)

	Skin problems	Fly and mosquito problems	Stomach problems	Malaria problems	Other problems
	(1)	(2)	(3)	(4)	(5)
Adopting six sanitation practices	-0.064*	-0.202***	-0.111***	-0.132***	-0.136
	(0.033)	(0.065)	(0.038)	(0.042)	(0.107)
Monthly expenditure per capita (ln)	0.012	-0.053	0.006	0.034	-0.044
	(0.029)	(0.037)	(0.023)	(0.034)	(0.051)
Female head	0.007	-0.044	-0.036**	-0.014	-0.073***
	(0.017)	(0.028)	(0.018)	(0.009)	(0.018)
Age head	-0.000	-0.001*	-0.000	0.000	0.000
	(0.000)	(0.001)	(0.000)	(0.000)	(0.001)
Household size	0.011***	0.000	0.023***	0.017***	0.007
	(0.004)	(0.006)	(0.006)	(0.004)	(0.008)
No. of males	-0.005	0.006	0.001	-0.008	-0.002
	(0.005)	(0.010)	(0.012)	(0.005)	(0.008)
No. of children	-0.006*	-0.012	-0.035***	0.002	-0.006
	(0.003)	(0.010)	(0.009)	(0.011)	(0.013)
No. of elders	-0.002	-0.010	-0.016	-0.008	0.021**
	(0.004)	(0.012)	(0.012)	(0.008)	(0.010)
Illiterate head	0.003	0.052***	-0.000	-0.023*	-0.031
	(0.012)	(0.017)	(0.015)	(0.013)	(0.039)
High school head	-0.015	-0.020	0.023	0.035**	-0.013
	(0.011)	(0.025)	(0.028)	(0.014)	(0.033)
Diploma head	0.005	0.075	-0.042	-0.067***	-0.133***
	(0.014)	(0.101)	(0.033)	(0.024)	(0.047)
Uni and above head	-0.022**	0.036	0.007	0.019	0.000
	(0.010)	(0.046)	(0.029)	(0.023)	(0.039)
Scheduled tribes	-0.032**	-0.165***	0.002	0.006	0.089
	(0.015)	(0.033)	(0.018)	(0.024)	(0.057)
Scheduled castes	-0.002	-0.031	0.010	-0.010	-0.005
	(0.006)	(0.024)	(0.014)	(0.012)	(0.014)
Hindu	0.017	-0.063	-0.040	-0.033	0.034
	(0.016)	(0.069)	(0.047)	(0.066)	(0.044)
Islam	0.048	-0.085	-0.030	-0.077	0.016
	(0.032)	(0.073)	(0.055)	(0.070)	(0.036)
Christianity	-0.004	-0.108	-0.041	-0.004	-0.076
	(0.016)	(0.102)	(0.090)	(0.073)	(0.069)
Sikhism	0.040*	-0.057	0.038	0.053	0.217***
	(0.024)	(0.074)	(0.060)	(0.052)	(0.068)
Buddhism	0.004	-0.278***	-0.060	-0.081	-0.179**
	(0.029)	(0.086)	(0.087)	(0.069)	(0.086)
constant	-0.017	1.122***	0.157	-0.110	0.669*
	(0.216)	(0.306)	(0.173)	(0.263)	(0.368)
Number of observations	10902	10902	10902	10902	10902
R^2	0.016	0.063	0.026	0.008	0.031
Adjusted R^2	0.014	0.061	0.025	0.006	0.030
Under-identification	0.009	0.009	0.009	0.009	0.009
Over-identification	0.424	0.668	0.203	0.613	0.786
Weak identification	176.479	176.479	176.479	176.479	176.479

Robust standard errors clustered at state levels; *** $p<0.01$, ** $p<0.05$, * $p<0.1$.

Supplementary Table S7G: Impacts of adopting a different number of sanitation practices on health problems: adopting more than six sanitation practices (IV regressions)

	Skin problems	Fly and mosquito problems	Stomach problems	Malaria problems	Other problems
	(1)	(2)	(3)	(4)	(5)
Adopting more than six sanitation practices	-0.077**	-0.237***	-0.104***	-0.098**	-0.100
	(0.033)	(0.067)	(0.039)	(0.040)	(0.070)
Monthly expenditure per capita (ln)	0.019	-0.039	-0.017	-0.007	-0.062
	(0.028)	(0.036)	(0.021)	(0.037)	(0.043)
Female head	0.015	-0.039	-0.036*	-0.015	-0.055***
	(0.018)	(0.030)	(0.019)	(0.009)	(0.020)
Age head	-0.000	-0.001	0.000	0.000	0.000
	(0.000)	(0.001)	(0.000)	(0.000)	(0.001)
Household size	0.012***	0.003	0.023***	0.014***	0.010
	(0.004)	(0.007)	(0.006)	(0.005)	(0.009)
No. of males	-0.005	0.008	-0.002	-0.006	-0.005
	(0.006)	(0.010)	(0.012)	(0.004)	(0.009)
No. of children	-0.010***	-0.017*	-0.032***	0.002	-0.004
	(0.003)	(0.010)	(0.010)	(0.011)	(0.011)
No. of elders	-0.002	-0.020	-0.025*	-0.008	0.012
	(0.004)	(0.013)	(0.013)	(0.008)	(0.008)
Illiterate head	-0.001	0.046***	-0.004	-0.024*	-0.034
	(0.014)	(0.018)	(0.016)	(0.013)	(0.038)
High school head	-0.018	-0.026	-0.015	0.025	-0.010
	(0.012)	(0.025)	(0.015)	(0.017)	(0.019)
Diploma head	0.010	-0.064	0.014	-0.077***	-0.058
	(0.018)	(0.109)	(0.032)	(0.026)	(0.077)
Uni and above head	-0.028***	0.052	-0.008	0.028	0.014
	(0.009)	(0.043)	(0.025)	(0.034)	(0.047)
ST caste	-0.029**	-0.161***	-0.003	-0.001	0.097*
	(0.015)	(0.032)	(0.018)	(0.024)	(0.056)
SC caste	-0.001	-0.043*	0.007	-0.011	-0.012
	(0.005)	(0.023)	(0.013)	(0.012)	(0.015)
Hindu	0.006	-0.053	-0.024	-0.004	0.074*
	(0.019)	(0.065)	(0.045)	(0.051)	(0.044)
Islam	0.045	-0.069	-0.012	-0.054	0.084**
	(0.038)	(0.069)	(0.051)	(0.054)	(0.042)
Christianity	0.015	-0.119	0.010	0.054	-0.042
	(0.020)	(0.107)	(0.093)	(0.057)	(0.072)
Sikhism	0.020	0.046	0.129*	0.120*	0.305***
	(0.033)	(0.069)	(0.066)	(0.063)	(0.062)
Buddhism	-0.027	-0.106	-0.054	-0.016	-0.070
	(0.020)	(0.099)	(0.079)	(0.055)	(0.080)
constant	-0.057	0.998***	0.308**	0.160	0.735**
	(0.206)	(0.307)	(0.137)	(0.293)	(0.335)
Number of observations	9846	9846	9846	9846	9846
R^2	0.013	0.050	0.023	0.013	0.024
Adjusted R^2	0.011	0.048	0.021	0.011	0.022
Under-identification	0.013	0.013	0.013	0.013	0.013
Over-identification	0.173	0.598	0.182	0.182	0.251
Weak identification	537.888	537.888	537.888	537.888	537.888

Robust standard errors clustered at state levels; *** $p<0.01$, ** $p<0.05$, * $p<0.1$.

Supplementary S8: Brief introduction of propensity score matching

The propensity score matching (PSM) method is first introduced by Rosenbaum and Rubin (1983). It is employed to ensure the similarity of the treatment and control groups in terms of observed baseline characteristics by balancing the two groups (Stuart et al., 2014). In the PSM, the matching algorithm using nearest neighbors is traditional (Cochran & Rubin, 1973; Dehejia and Wahba 2002), hence, we employed it in our estimation. First, a Probit model is run to estimate the propensity scores as:

$$P(SA_i = 1|X_i, S_k) = G(\alpha_0 + \alpha_1 X_i + \alpha_2 S_k) \quad \text{(A1)}$$

In Equation (A1), SA_i captures the adopted practices of household *i* and can be either *SA_single* (as adopting only specific practice or *SA_bundle* (as adopting one, two… or more than six practices). X_i are the vector of household characteristics which include (i) demographic characteristics of the household head and the household (i.e., age, gender, and education of household head; household size, number of males, number of children, and number of elders), (ii) wealth status (i.e., monthly per capital consumption), (iii) social groups (i.e., scheduled castes or scheduled tribes), and (iv) religions (i.e., Hindu, Islam, Christianity, Sikhism, and Buddhism); S_k is the dummies for states.

The treatment effect of the treated (TT) *i* on health issue (H_i) can be calculated as:

$$\widehat{TT_i} = H_i SA_i - \sum_{j\in C_i} w_{i,j} H_j (1 - SA_{j)} \quad \text{(A2)}$$

Then, the average treatment effect on the treated (ATT) can be calculated as:

$$\widehat{ATT} = ave(\widehat{TT_i}|SA_i = 1) \quad \text{(A3)}$$

Cited documents:

Cochran, W. G., & Rubin, D. B., (1973). Controlling bias in observational studies: A review. *Sankhyā: The Indian Journal of Statistics, Series A*, 417-446. www.jstor.org/stable/2504989. Accessed January 20, 2025.

Dehejia, R. H., & Wahba, S., 2002. Propensity score-matching methods for nonexperimental causal studies. *Review of Economics and Statistics*, *84*(1), 151-161. https://doi.org/10.1162/003465302317331982

Rosenbaum, P. R., & Rubin, D. B., (1983). The central role of the propensity score in observational studies for causal effects. *Biometrika*, *70*(1), 41-55. https://doi.org/10.1093/biomet/70.1.41

Stuart, E. A., Huskamp, H. A., Duckworth, K., Simmons, J., Song, Z., Chernew, M. E., & Barry, C. L., (2014). Using propensity scores in difference-in-differences models to estimate the effects of a policy change. *Health Services and Outcomes Research Methodology*, *14*(4), 166-182. https://doi.org/10.1007/s10742-014-0123-z

Supplementary Table S8A: The Average Treatment on the Treated (ATT) of adopting only one specific sanitation practice on health problems

		Skin problems	Fly and mosquito problems	Stomach problems	Malaria problems	Other problems
		(1)	(2)	(3)	(4)	(5)
Drinking water (tap)	Treated (yes)	0.042	0.542	0.056	0.111	0.111
	Controls (no)	0.039	0.461	0.142	0.103	0.239
	Difference	0.003	0.081	-0.086**	0.008	-0.128***
	Std. Err.	0.027	0.069	0.037	0.043	0.048
Non-drinking water (tap)	Treated (yes)	0.043	0.674	0.196	0.196	0.283
	Controls (no)	0.104	0.557	0.13	0.113	0.348
	Difference	-0.061	0.117	0.065	0.083	-0.065
	Std. Err.	0.04	0.081	0.066	0.065	0.078
Treated drink- water	Treated (yes)	0.064	0.441	0.188	0.17	0.355
	Controls (no)	0.063	0.468	0.172	0.152	0.376
	Difference	0.001	-0.027	0.016	0.019	-0.02
	Std. Err.	0.011	0.021	0.017	0.016	0.021
Wash hand before meal with soap	Treated (yes)	0.092	0.618	0.163	0.145	0.31
	Controls (no)	0.105	0.558	0.208	0.132	0.395
	Difference	-0.013	0.060***	-0.045***	0.012	-0.084***
	Std. Err.	0.013	0.021	0.016	0.015	0.02
Toilet (exclusive use)	Treated (yes)	0.1	0.557	0.178	0.113	0.409
	Controls (no)	0.099	0.55	0.197	0.124	0.397
	Difference	0.001	0.007	-0.019**	-0.011	0.012
	Std. Err.	0.007	0.011	0.009	0.008	0.011
Bathroom (exclusive use)	Treated (yes)	0.069	0.526	0.202	0.13	0.296
	Controls (no)	0.083	0.566	0.201	0.141	0.338
	Difference	-0.015	-0.040*	0.001	-0.011	-0.042**
	Std. Err.	0.011	0.021	0.017	0.014	0.019
Drainage (underground or covered pucca)	Treated (yes)	0.118	0.656	0.153	0.118	0.307
	Controls (no)	0.111	0.631	0.222	0.16	0.354
	Difference	0.008	0.025	-0.069***	-0.042***	-0.047**
	Std. Err.	0.015	0.022	0.017	0.015	0.022
Arranged garbage collection	Treated (yes)	0.081	0.532	0.146	0.107	0.32
	Controls (no)	0.068	0.507	0.146	0.099	0.286
	Difference	0.013	0.025	0.000	0.008	0.034
	Std. Err.	0.013	0.023	0.017	0.015	0.022

Nearest Matching (5 nearest neighbors; Caliper = 0.02, Common support); *** $p<0.01$, ** $p<0.05$, * $p<0.1$.

Supplementary Table S8B: The Average Treatment on the Treated (ATT) of households adopting one specific sanitation practice along with at least another practice on health problems

		Skin problems	Fly and mosquito problems	Stomach problems	Malaria problems	Other problems
		(1)	(2)	(3)	(4)	(5)
Drinking water (tap)	Treated (yes)	0.042	0.379	0.1	0.091	0.248
	Controls (no)	0.065	0.432	0.174	0.12	0.323
	Difference	-0.023	-0.054*	-0.074***	-0.029	-0.076***
	Std. Err.	0.017	0.03	0.023	0.021	0.029
Non-drinking water (tap)	Treated (yes)	0.046	0.358	0.113	0.1	0.273
	Controls (no)	0.088	0.447	0.208	0.112	0.348
	Difference	-0.042**	-0.089**	-0.094***	-0.012	-0.075**
	Std. Err.	0.02	0.036	0.028	0.025	0.035
Treated drink- water	Treated (yes)	0.057	0.409	0.156	0.105	0.322
	Controls (no)	0.09	0.431	0.182	0.12	0.384
	Difference	-0.033**	-0.022	-0.026	-0.015	-0.062**
	Std. Err.	0.016	0.03	0.024	0.022	0.03
Wash hand before meal with soap	Treated (yes)	0.062	0.429	0.14	0.098	0.296
	Controls (no)	0.094	0.491	0.209	0.126	0.358
	Difference	-0.032**	-0.062**	-0.068***	-0.028	-0.062**
	Std. Err.	0.016	0.026	0.021	0.018	0.026
Toilet (exclusive use)	Treated (yes)	0.068	0.441	0.149	0.105	0.329
	Controls (no)	0.09	0.497	0.193	0.126	0.364
	Difference	-0.022**	-0.056***	-0.045***	-0.021	-0.035*
	Std. Err.	0.011	0.018	0.015	0.013	0.018
Bathroom (exclusive use)	Treated (yes)	0.064	0.426	0.141	0.101	0.313
	Controls (no)	0.084	0.49	0.187	0.124	0.351
	Difference	-0.02	-0.065***	-0.045***	-0.023	-0.039*
	Std. Err.	0.012	0.02	0.016	0.014	0.02
Drainage (underground or covered pucca)	Treated (yes)	0.065	0.447	0.138	0.108	0.305
	Controls (no)	0.075	0.505	0.168	0.144	0.303
	Difference	-0.01	-0.058**	-0.03	-0.036**	0.003
	Std. Err.	0.014	0.023	0.019	0.016	0.023
Arranged garbage collection	Treated (yes)	0.046	0.432	0.123	0.085	0.319
	Controls (no)	0.064	0.48	0.162	0.102	0.277
	Difference	-0.018	-0.049**	-0.039**	-0.017	0.042*
	Std. Err.	0.013	0.022	0.017	0.015	0.021

Nearest Matching (5 nearest neighbors; Caliper = 0.02, Common support); *** p<0.01, ** p<0.05, * p<0.1.

Supplementary Table S8C: The Average Treatment on the Treated (ATT) of households adopting a different number of sanitation practices on health problems

		Skin problems	Fly and mosquito problems	Stomach problems	Malaria problems	Other problems
		(1)	(2)	(3)	(4)	(5)
Adopting one sanitation practice	Treated (yes)	0.092	0.549	0.176	0.123	0.373
	Controls (no)	0.094	0.543	0.185	0.134	0.376
	Difference	-0.002	0.006	-0.009	-0.01	-0.003
	Std. Err.	0.005	0.009	0.007	0.006	0.009
Adopting two sanitation practices	Treated (yes)	0.079	0.501	0.171	0.123	0.361
	Controls (no)	0.092	0.528	0.187	0.138	0.367
	Difference	-0.012*	-0.027**	-0.015	-0.015*	-0.006
	Std. Err.	0.007	0.012	0.01	0.008	0.012
Adopting three sanitation practices	Treated (yes)	0.07	0.465	0.152	0.106	0.323
	Controls (no)	0.083	0.501	0.178	0.127	0.345
	Difference	-0.013	-0.036**	-0.026**	-0.021*	-0.022
	Std. Err.	0.01	0.017	0.013	0.012	0.017
Adopting four sanitation practices	Treated (yes)	0.062	0.411	0.132	0.094	0.304
	Controls (no)	0.076	0.457	0.16	0.129	0.327
	Difference	-0.015	-0.047**	-0.029	-0.035**	-0.022
	Std. Err.	0.013	0.023	0.018	0.016	0.023
Adopting five sanitation practices	Treated (yes)	0.05	0.386	0.134	0.09	0.303
	Controls (no)	0.067	0.448	0.179	0.127	0.331
	Difference	-0.017	-0.062**	-0.045*	-0.037*	-0.029
	Std. Err.	0.017	0.031	0.023	0.022	0.03
Adopting six sanitation practices	Treated (yes)	0.035	0.336	0.1	0.087	0.256
	Controls (no)	0.076	0.424	0.191	0.121	0.344
	Difference	-0.041**	-0.088**	-0.090***	-0.034	-0.088**
	Std. Err.	0.019	0.039	0.028	0.027	0.037
Adopting more than six sanitation practices	Treated (yes)	0.041	0.358	0.102	0.086	0.252
	Controls (no)	0.087	0.43	0.205	0.126	0.352
	Difference	-0.045**	-0.072	-0.102***	-0.04	-0.100**
	Std. Err.	0.023	0.044	0.034	0.03	0.042

Nearest Matching (5 nearest neighbors; Caliper = 0.02, Common support); *** p<0.01, ** p<0.05, * p<0.1.

Supplementary Table S8D: Covariate balancing tests for propensity score matching (average treatment on the treated)

	Matching	Pseudo-R2	LR chi2	p>chi2	Mean Bias	Med Bias	B	R	% concern	% bad
Adopting only one specific sanitation practice										
Drinking water (tap)	Before	0.066	56.400	0.000	15.9	10.2	89.5*	0.73	17	22
	After	0.089	1078.240	0.000	13.7	15.0	70.1*	0.35*	11	28
Non-drinking water (tap)	Before	0.073	41.800	0.000	19.2	15.5	86.6*	0.32*	33	17
	After	0.109	1257.240	0.000	10.0	5.9	79.6*	0.63	39	17
Treated drink- water	Before	0.068	541.770	0.000	10.7	6.1	66.6*	1.5	28	17
	After	0.029	621.230	0.000	8.7	7.6	40.3*	0.97	28	17
Wash hand before meal with soap	Before	0.020	103.140	0.000	7.6	7.3	38.5*	1.11	17	11
	After	0.003	61.020	0.000	3.1	3.3	12.6	1.2	22	0
Toilet (exclusive use)	Before	0.046	997.590	0.000	9.6	6.3	51.4*	1.2	33	6
	After	0.001	13.280	0.775	1.0	0.7	5.8	1.06	11	0
Bathroom (exclusive use)	Before	0.044	258.690	0.000	9.9	7.6	56.6*	1.11	22	11
	After	0.020	433.310	0.000	6.0	3.0	33.5*	1.57	17	11
Drainage (underground or covered pucca)	Before	0.053	246.730	0.000	11.9	10.6	65.7*	0.48*	28	11
	After	0.014	271.200	0.000	4.6	3.3	28.0*	1.05	11	11
Arranged garbage collection	Before	0.043	264.960	0.000	10.8	6.7	55.0*	1	28	0
	After	0.003	58.700	0.000	1.9	1.3	12.3	1.22	11	0
Adopting one specific sanitation practice along with at least another practice										
Drinking water (tap)	Before	0.331	8265.490	0.000	25.9	22.9	150.8*	1.75	28	33
	After	0.003	73.470	0.000	2.3	1.6	13.8	0.91	11	0
Non-drinking water (tap)	Before	0.329	8002.070	0.000	26.1	23	150.3*	1.75	28	33
	After	0.004	80.390	0.000	1.9	1.6	14.4	1.02	17	11
Treated drink- water	Before	0.291	9757.680	0.000	28.2	22.9	143.5*	2.04*	33	33
	After	0.016	326.790	0.000	5.7	4.9	29.9*	1.88	22	0
Wash hand before meal with soap	Before	0.223	9593.560	0.000	24.2	20.7	129.2*	1.76	22	33
	After	0.007	147.270	0.000	4.4	4.6	20	1.45	22	0
Toilet (exclusive use)	Before	0.223	9593.560	0.000	24.2	20.7	129.2*	1.76	22	33
	After	0.001	30.120	0.036	1.4	0.9	8.7	1.33	6	0
Bathroom (exclusive use)	Before	0.268	10836.160	0.000	26.1	23	141.0*	1.68	28	33
	After	0.004	78.200	0.000	1.9	0.9	14	1.53	6	0
Drainage (underground or covered pucca)	Before	0.312	8310.830	0.000	27	22.2	146.3*	1.75	22	39
	After	0.007	148.600	0.000	4.4	5.4	19.2	2.05*	17	0
Arranged garbage collection	Before	0.280	7511.710	0.000	23.8	19.5	136.5*	1.86	22	33
	After	0.004	85.350	0.000	2.5	2.1	14.7	1.04	22	0
Adopting a different number of sanitation practices										
Adopting one sanitation practice	Before	0.032	867.580	0.000	7.5	5.1	42.7*	1.05	39	6
	After	0.000	5.860	0.997	0.5	0.5	3.8	1.01	6	0
Adopting two sanitation practices	Before	0.112	3077.310	0.000	15.5	12.1	82.3*	1.22	22	22
	After	0.001	18.400	0.429	1.0	0.5	6.8	1.24	11	0
Adopting three sanitation practices	Before	0.227	5965.200	0.000	22.5	17.3	121.6*	1.52	17	33
	After	0.008	182.660	0.000	3.3	2.5	21.3	1.8	11	0
Adopting four sanitation practices	Before	0.324	7423.280	0.000	26.9	21.2	148.4*	1.84	28	33
	After	0.019	418.920	0.000	5.5	3.7	31.8*	3.09*	28	0
Adopting five sanitation practices	Before	0.389	7292.840	0.000	29.2	24.3	166.0*	1.77	28	33
	After	0.027	583.270	0.000	9.6	7.2	39.0*	1.95	33	6
Adopting six sanitation practices	Before	0.429	5461.950	0.000	30.6	26.8	178.7*	1.78	28	33
	After	0.157	3074.680	0.000	19.9	16.3	100.2*	1.07	72	6
Adopting more than six sanitation practices	Before	0.520	5008.050	0.000	35.3	29.9	210.2*	1.61	33	33
	After	0.130	1756.570	0.000	13.6	9.4	88.4*	0.59	44	39

* if B>25%, R outside [0.5; 2]; B is the standardized difference in the means of the propensity scores between treated and controlled households; R the ratio of the variances of the propensity scores for treated and controlled households; Nearest Matching (5 nearest neighbors; Caliper = 0.02, Common support).
